# Analyzing the digital customer journey: a novel framework for sequential behavior modeling

**DOI:** 10.1038/s41598-026-43762-8

**Published:** 2026-03-23

**Authors:** Lihi Bar-El, Hila Chalutz-BenGal, Sivan Gazit, Tal Patalon, Irad Ben-Gal

**Affiliations:** 1 School of Industrial & Intelligent Systems Engineering, Tel Aviv University, 69978 Ramat Aviv, Tel Aviv, Israel; 2 The Alexander Kofkin Faculty of Engineering, Bar-Ilan University, 5290002 Ramat-Gan, Israel; 3 Maccabi Healthcare Services (MHS), 27 HaMered Street, 6812509 Tel Aviv, Israel

**Keywords:** Digital customer journey, Event-log processing, Healthcare application, Machine learning, People analytics, Sequential behavior modeling, Computational biology and bioinformatics, Health care, Mathematics and computing

## Abstract

In recent years, the growing amount of event-log data collected from websites and mobile applications has provided an opportunity to analyoc2vec to cluster physical ze user-behavior patterns in digital customer systems. However, a holistic data-driven approach for analyzing the digital customer journey is lacking. To address this gap, we propose a method for analyzing large amounts of digital event logs to derive actionable insights related to customers’ digital journeys, in order to improve user experience, satisfaction, and loyalty, particularly in high-stakes domains like healthcare. The proposed method identifies the most relevant types of customer journeys via the latent Dirichlet allocation (LDA) algorithm and its hierarchical version (HLDA). The proposed method is executed by generating topics from sequences of events that are transformed into session clusters. We then capture sequential behavioral patterns among and within the clusters and further model them as Markov chains. The results of this method for various applications are presented. We demonstrate the most representative paths and detect customers’ actions with a high probability of potential undesirable outcomes. We utilize a unique real-world dataset of event logs extracted from a mobile application of a large health maintenance organization (HMO). This dataset includes over 120,000 active customers who generated more than 5.5 million events per day. This research makes three main contributions: )i) we propose a novel methodology that integrates established techniques (LDA/hLDA, Markov chain analysis, and leakage/irregularity detection) for analyzing digital customer journeys at various granularity levels to simplify the variability of the customer journey; (ii) we show how topic-based session summarization reduces complexity and supports customer journey’s sequential diagnostics, for example by detecting irregularities of unique behavioral patterns that would otherwise be difficult to detect; and (iii) we demonstrate the framework on a unique real-world HMO dataset that enables the implementation of the method to extract actionable and updated insights important for the digital healthcare domain. Finally, we discuss the emerging role of Artificial Intelligence and Large Language Models in digital customer journey analysis.

## Introduction

Customer experience improvement has recently attracted the attention of scholars and practitioners^1,2^ with the aim of determining how the various interactions a customer has with a company or service contribute to the overall experience and serviceability and strengthen customer loyalty. A digital customer journey is defined by a sequence of steps that customers go through when interacting with a digital system while trying to accomplish a certain task. These sequences are typically referred to as “sessions.”

With the rise of digital services, many companies in diverse industries, such as e-commerce and e-banking, are prioritizing improvements in service quality and the customer journey^3–5^. As a result, these companies often gather and store large volumes of customer interaction data in the form of event logs. Furthermore, as services become more complex, the numbers of feasible journeys, interactions, and event logs undergo a significant surge, leading to the emergence of what is commonly referred to as a “spaghetti model.” This situation poses a substantial challenge when attempting to analyze customer journeys and extract meaningful insights regarding customer behavior patterns^4,6–8^. Consequently, it is imperative to employ models that are capable of reducing the multitude of potential journeys through techniques such as clustering, thereby grouping the journeys into a reasonable number of representative categories. Additionally, creating summaries of event sequence data^9^ and addressing the escalating length of such sequences^10^ are crucial endeavors. While the investigation of sequential patterns proves more enlightening than examining individual actions^11^, opting to encompass the entire spectrum of potential routes without implementing any form of route reduction exposes us to the risk of developing models characterized by an exponentially expanding array of states and parameters, consequently leading to considerable computational complexity. Other challenges are related to the fact that, in most cases, a person with domain expertise must be involved to produce a high-quality analysis. In addition, the data are only partially observed, which implies that one does not know exactly what patterns are relevant, making it difficult to evaluate the results.

Notably, a meaningful pattern of customer behavior should reveal more than just what actions and how many actions customers take during a visit to a digital system; it should enable us to infer customers’ intentions when accessing the system and understand whether they reached their objective and, if not, what factors prevented them from reaching it. In this way, one can gain actionable insights and, in certain cases, even analyze how these patterns differ across various customer characteristics^6^.

The ability to understand digital customer behavior can play an important role in detecting gaps between planned journeys and actual journeys reflected by the data logs^7^. Early detection of such gaps can prevent undesirable consequences, such as customer churn, inefficient journeys and repeated technical errors. These insights can be crucial for improving service quality and reducing customer frustration. Improving the customer journey is also a key factor in reducing operational costs, such as when customers switch to digital rather than physical services (e.g., call centers), resulting in a reduced need for manpower.

In this work, we propose a framework for customer journey analysis to leverage digital event log data that are aggregated into sessions. The first phase aims to group sessions into an appropriate number of clusters. These clusters represent the main types of journeys through which customers travel. As shown later in a series of experiments, latent Dirichlet al.location (LDA)^12^ has been found to be an efficient clustering method for the considered task in terms of two clustering evaluation metrics. Originally, LDA was used for natural language processing (NLP) to analyze large text corpora. We follow previous works and treat each action (e.g., clicking a selection) as a word and each stream of such actions as a virtual sentence. A significant output of LDA is the generated “topics”, which facilitate the interpretation of the obtained clusters. The second phase aims to capture sequential behaviors in each cluster by analyzing them via Markov models. We show that even a simple first-order Markov model is computationally manageable and able to find the main pattern of subsequent actions. We then extend the first-order Markov model to a higher-order Markov model and show how more complex patterns can be extracted. This step is necessary for further analysis, such as finding the most representative paths in each cluster or identifying which actions lead to undesirable outcomes (e.g., errors).

The described structure of our methodology, which starts with clustering sessions and then applies Markov models, has several advantages. First, it enables us to reduce the number of model parameters in the Markov models since the obtained clusters are homogeneous and require a small number of actions to represent. This process also reduces the sparsity of the Markov models compared with that of the same models when the clustering phase prior to construction is excluded.

Second, with respect to space complexity, our approach does not require the construction of a pairwise session distance matrix. Typically, the number of sessions in digital event logs is extremely large, making this task practically infeasible^8^.

By combining LDA and Markov models, we discover unique and unexpected behaviors that cannot be identified via a single method. We do this by showing the discrepancies between LDA and Markov models in terms of cluster outcomes; for example, LDA assigns a session to one cluster, but the Markov model assigns it to a different cluster. These cases may indicate ambiguity in the task being performed in the session (e.g., when multiple tasks occur in a single session). When such irregular behaviors are detected, they can be further investigated, as shown later.

The contribution of this work is threefold. First, we present a systematic, end-to-end framework for digital customer journey analysis that integrates established methods in a reproducible pipeline to detect dominant journey types and derive actionable insights. Second, we show how topic modeling–based session summarization (such as LDA and hLDA) can be combined with Markov chain–based sequence analysis to capture both what users do (salient actions within journey types) and how they move between actions (transition dynamics). We also leverage hLDA to support multi-granularity journey analysis, enabling journey types to be examined at different abstraction levels. Third, we demonstrate the framework on a large-scale real-world dataset from a smart health application, highlighting practical considerations for preprocessing, scalability, and interpretability.

The remainder of this paper is organized as follows. Section “Related work” presents related work. Section “Proposed approach: digital customer journey analysis framework” introduces the proposed methodology for analyzing customer journeys. Section “Results on real-world data” illustrates the implementation of the proposed methodology using a real dataset. Finally, Section “Discussion, contributions and future work” concludes the work and discusses future research directions.

## Related work

The literature presents four main theoretical concepts that are associated with factors for addressing the methodology proposed in the next section. Analyzing the current literature, this study identifies a significant gap in the literature for a systematic approach to analyzing customer journeys that goes beyond mere visualization to effectively capture customer behavior^5^. Additionally, we note a shortage of methods in the literature that are capable of efficiently processing vast amounts of event log data while maintaining manageable space and computational costs.

### Customer journey mapping (CJM)

Most of the work related to customer journeys has focused on customer journey mapping (CJM)^13^, a method for summarizing and visualizing the actual paths customers take when interacting with a company or service. The main challenge in CJM is to reduce the increasing number of possible paths to a reasonable number that is easy for people to visualize and interpret (referring mainly to the “spaghetti model” mentioned above). Many variants of methods have been presented in the literature to overcome this challenge.

In most previous papers, various types of conventional clustering algorithms, such as K-means^14,15^, variations of K-means^16^ and hierarchical clustering^17^, were used to find the representative groups of digital customer journeys over the recorded sessions. An essential component of these algorithms is the representation sessions, which are usually variable-length sequences.

In Fujimoto et al.^1^, researchers used word embedding techniques such as TF-IDF to represent web sessions as fixed-size vectors. Others^18^ used doc2vec to cluster physical navigations, even though it is still used mostly for document clustering, such as in^19,]^^20^. A different approach is to leave sessions unchanged but instead define a distance measure between pairs of sessions. An example is the work of Bernard and Andritsos^12^, who presented a web tool for interactive journey exploration. This tool is based on a hierarchical clustering algorithm embedded over the shingle distance metric, which is a variation of the Jaccard distance. In Koltcov et al.^21^, the authors proposed VLVD, another variation of the Jaccard distance that can handle variable session lengths. A major drawback of these methods is that they handle each element in the session independently of its context; thus, they do not capture the internal order of the sequences, which is crucial for understanding digital navigational patterns.

To address this problem, researchers have proposed an interplay between hierarchical clustering and Markov models, where each session is represented as a Markov model, thereby accounting for the internal order of the sessions. This type of sequence representation has been used for clustering both physical^22^ and digital^23^ journeys. In Harbich et al.^24^, Markov models were used as a single method for clustering sessions, while clusters were modeled as a mixture of K Markov models, and an optimization phase was implemented via expectation-maximization (EM)^25^.

CJM has proven to be a useful tool that provides valuable insights into customer activities. However, its main drawback is that it displays the data “as is” without the ability to understand the underlying patterns of customer behavior. Therefore, a deep analysis of customer journeys is needed.

### Customer journey analysis (CJA)

Although there are numerous works on CJM in the literature, there are only a few works that thoroughly analyze journeys, which is known as customer journey analysis (CJA). CJA takes CJM one step further and aims to capture, measure, analyze, and evaluate the quality and outcome of the customer experience^26,27^. As Halvorsrud et al.^12^ noted, few works have addressed the problem of formalizing a repeatable methodology for analyzing customer journeys [27; 53]. Even fewer works have addressed the challenge of developing a data-driven framework for CJA. In the digital world, this type of analysis is often referred to as “web usage mining”, i.e., the automatic discovery and analysis of meaningful patterns and relationships from a large collection of semi-structured data, such as clickstream data^28^.

This process often involves three steps:

1. *Data collection and preprocessing*—conversion of raw data into a format that can be easily and effectively processed by statistical models and ML tools (e.g., aggregation of event log data into session-level data);
2. *Pattern discovery*—the discovery of hidden patterns that represent key patterns of customer behavior^6^;
3. *Pattern analysis*—further filtering and processing of patterns found in step 2 to produce meaningful insights (e.g., analysis of relationships between patterns).

An application of web usage mining was presented by Raphaeli et al.^29^, who used sequential association rule mining to identify differences between online consumption behavior on mobile devices and that on PCs. The authors showed how insights can be derived from the proposed methodology, such as the finding that mobile sessions are more task-oriented, whereas PC sessions are more exploratory.

Terragni & Hassani^30,31^ presented repeatable frameworks for CJA by process mining. They showed how web log data from online customer journeys can be analyzed to recommend web pages in a personalized manner.

Our proposed framework follows the web usage mining methodology^4^, as will be described in the next sections.

### Topic modeling

Topic modeling is a type of statistical algorithm for detecting abstract “topics” that occur in a collection of documents^32^. Topic modeling is often used as a text-mining tool to discover hidden semantic structures in a text. One of the best-known topic modeling algorithms is LDA.

### Latent Dirichlet allocation (LDA)

LDA is a generative statistical model that was originally introduced and developed by Blei^33^ for characterizing text documents. In this model, each document is represented as a multinomial distribution of topics, and each topic is represented as a multinomial distribution of words.

LDA has also been extended to collections of discrete data other than documents. In the context of customer journeys, it has been used to find physical navigation patterns^34^ and to detect latent clinical pathways^35^. In Xu et al.^36^, it was shown that LDA can improve the performance of website recommender systems by feeding LDA session topic distribution results into these systems. Fujimoto et al.^1^ showed that LDA can be successfully used for web customer profiling and clustering. One of the greatest advantages of LDA is its ability to process large datasets^11^. In the proposed framework, LDA is used to identify co-occurrence-based latent task structures and reduce the dimensionality of the event-log space prior to sequential modeling. Temporal order is subsequently modeled utilizing Markov chains. As a result, topic modeling and sequence modeling serve complementary roles within the framework. The fact that LDA performs very well for documents, which typically contain more possible words in a document compared to possible actions in a session, suggests that it has the potential to perform well when applied to session data.

In the next section, we elaborate on how text data can be mapped to session data via LDA.

### Hierarchical Latent Dirichlet allocation

Hierarchical LDA (hLDA) is an extension of LDA that was introduced in Blei et al.^37^. hLDA is an algorithm that applies the nested Chinese restaurant process (nCRP)^38^ to LDA, where the latent topics found in a set of documents are structured hierarchically in a tree with L levels. Each node in the tree represents a topic, and each level in the tree represents a different level of topic abstraction: the higher a topic is in the hierarchy, the more abstract it is. The main advantage of hLDA is that both the topics and the *structure* of the tree are learned from the observed data; there is no need to specify the structure of the tree in advance.

hLDA has been used in several domains other than text^39^, e.g., for action recognition in videos^40^ and for identifying the latent characteristics behind frame-level musical features^41^. To our knowledge, hLDA has not been used for event log data, especially for digital customer journeys. In this context, topics represent the main tasks that the customer tries to accomplish during a session, and hLDA can be used to represent all journeys in a tree with different levels of granularity. This implies that the root of the tree represents a topic that spans all sessions, whereas topics at lower levels of the tree become more specific. It is important to note that LDA/hLDA do not preserve temporal order within sessions. Rather than modeling sequence dynamics at this stage, the objective of topic modeling is to identify homogeneous groups of sessions based on action co-occurrence^37,39^. This abstraction step reduces noise and sparsity before applying sequence models. Sequential dependencies are then modeled in the second stage via cluster-specific Markov chains, as explained next.

### Markov chains

A Markov chain is a stochastic model that describes a sequence of possible events, in which the probability of each event depends solely on the state attained in the previous events. Markov chains provide an effective and compact way to represent event log data. The fact that Markov chains consider the order of events makes them useful for different tasks in the world of digital customer journeys. Harbich et al.^24^ used a mixture of Markov models to create a customer journey mapping, whereas Marques et al.^23^ showed how Markov chains can be used to discover student profiles on a web-based e-learning platform.

In summary, we have identified a need in the literature for a systematic method for analyzing customer journeys that can capture customer behavior, derive actionable insights from it and not simply visualize the journeys. We have also found that methods that can process large amounts of event log data with reasonable space complexity and computational costs are lacking. We address these gaps by proposing a reproducible framework that can draw a complete picture of digital behavior while reducing runtime and space complexity compared with previous methods.

## Proposed approach: digital customer journey analysis framework

**Fig. 1:**
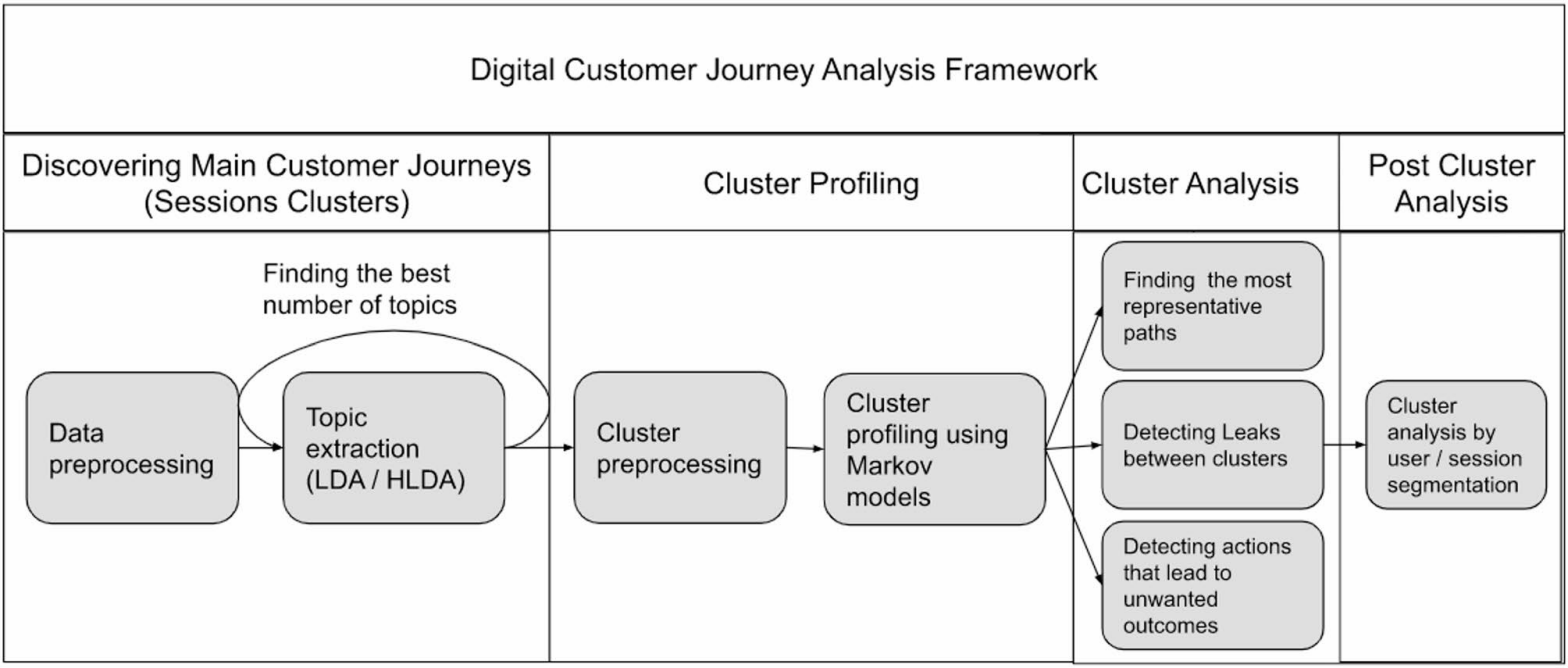
Digital customer journey analysis framework.

A general view of the proposed method and its main phases is presented below and sketched in Fig. 1:

1. Discovering the main customer journeys.
  - Data preprocessing: Data cleaning and aggregation of the raw data from event logs into sequences (sessions) are performed.
  - Deriving topics from the obtained sequences via LDA has been shown to be the preferred method. In this part, an optimization phase is implemented to select the best number of topics, and LDA generates topics on the basis of this number.
2. Cluster profiling.
  To derive sequential behavior patterns, we represent each topic as a Markov model. Since the LDA results correspond to the soft clustering results (each session has a probability of being assigned to each of the topics), this part aims to generate hard clusters from the obtained topics.
  - The LDA results, which correspond to soft clustering outputs, are converted into hard clusters by assigning each session to its dominant topic, defined as the topic with the highest posterior probability. This operationalization follows directly from the probabilistic structure of LDA and is widely used when downstream sequence modeling requires discrete cluster membership. While this conversion entails a partial loss of probabilistic information, it substantially improves interpretability and reduces computational complexity when constructing cluster-specific Markov models. Importantly, this step is not mandatory; weighted Markov models based on session-topic probabilities could alternatively be constructed.
  - Once clusters are created, a first-order or higher-order Markov model is established for each of the obtained clusters.
  Note that converting topics to hard clusters is optional since the Markov models can also be built using soft clustering results (e.g., by building weighted Markov models based on the session probabilities assigned to the topics). The advantage of the hard-clustering approach is that one immediately gains a clear picture of the clusters obtained, with a minimum number of computations, based on the sessions assigned to them. In a later stage of the proposed methodology, one can analyze leaks between clusters to recover some errors related to the hard-clustering stage.

3. Cluster analysis—The obtained Markov models are used to analyze behavioral patterns within clusters:
  - The most representative paths in each cluster are identified.
  - Leaks between clusters are defined as sessions exhibiting high posterior probability across multiple topics or inconsistencies between topic-based assignment and the transition dynamics implied by the Markov model. Such cases reflect behavioral ambiguity, suggesting that multiple tasks may occur within a single session. Rather than representing noise, these observations provide meaningful signals regarding complex user intent.
  - Actions that lead to unwanted outcomes (e.g., technical errors or ending sessions without finishing the requested task) are detected.
4. Post cluster analysis.

The goal of this phase is to enrich the cluster analysis with additional information (e.g., customer/session characteristics) and determine how the analyses in phase 3 are affected by different segments. Such segments can be related, for example, to the customer age, sociodemographic score, geographic location, device used and other factors.

In the following sections, we present the framework and its methods in detail.

### Discovering the main customer journeys

The first step of the proposed framework involves discovering the main customer journeys from event log data. This part includes the stages listed below.

#### Data preprocessing

**Fig. 2:**
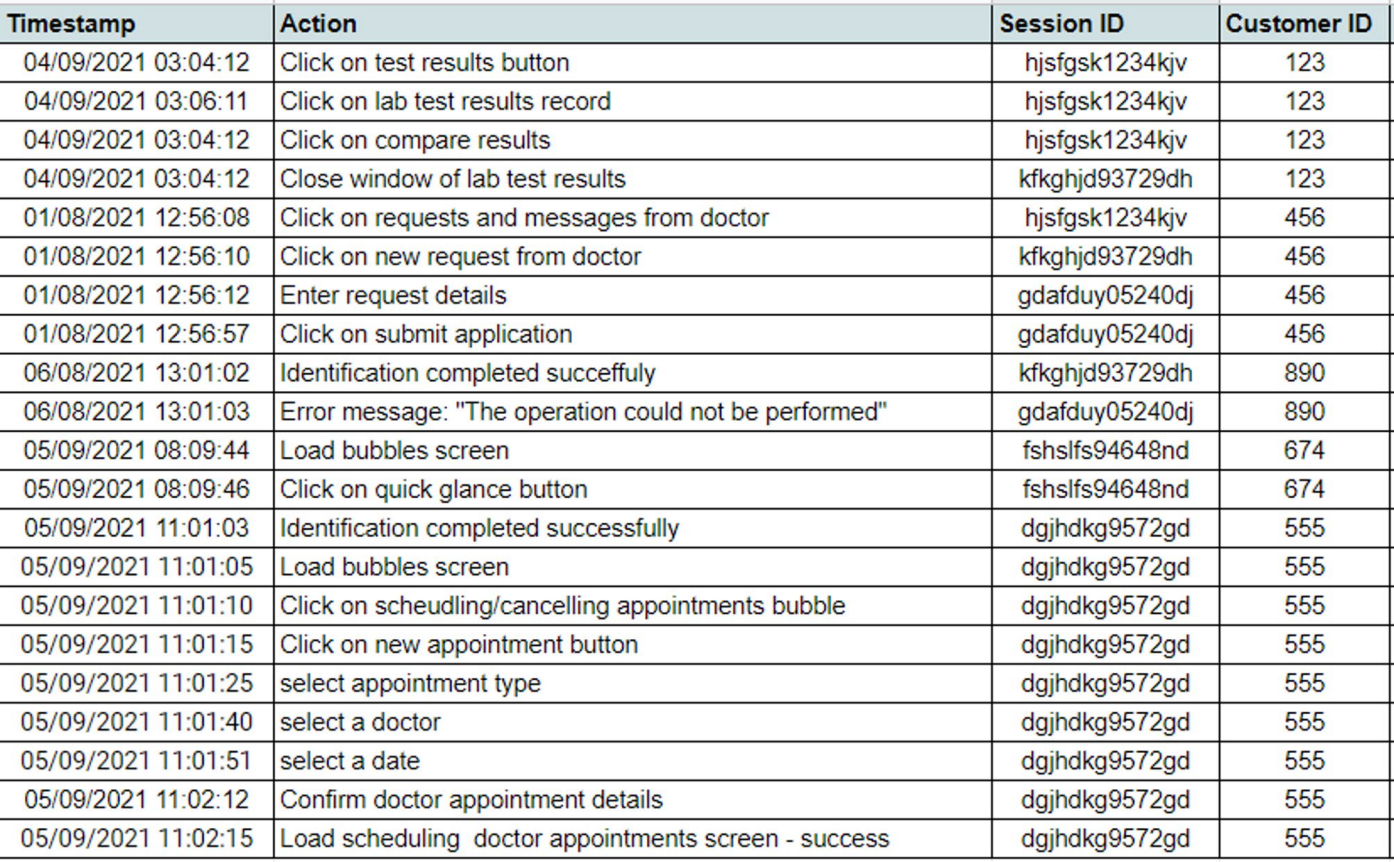
Example of event log data.

Data preprocessing is an essential component of the methodology and aims to convert the raw data from event logs into a form that is suitable for the LDA algorithm. Each event log should contain a timestamp, a description of the event (usually a string), the customer ID and, ideally, a session ID, as shown in Fig. 2. If no session ID exists, it should be created. Although this topic is beyond the scope of this work, several works have addressed the creation of such session identifiers. The most common approach is to set a threshold of 30 min for the inactivity time between two consecutive sessions^42,43^. Although the empirical demonstration in this study focuses on a healthcare mobile application, the proposed preprocessing phase is applicable to any digital environment that generates event logs with at least a timestamp, a user identifier, and a session identifier (or sufficient information to reconstruct sessions). The main domain-specific choices in our implementation concern (i) the minimum and maximum session lengths used for filtering extreme sessions and (ii) the manual exclusion of overly frequent, low-information actions (for example, mandatory login steps), which can be adapted in other domains based on their own usage patterns and business rules.

##### Data sequencing

All actions that occur during a single session (of a single customer) are combined into a sequence of actions in chronological order according to the step when they are executed.

##### Session length filtering

Sessions that were extremely short (e.g., containing fewer than two meaningful actions) were excluded, as they do not contain sufficient behavioral information for clustering or transition modeling. Similarly, sessions exceeding the 99th percentile in length were removed to mitigate the influence of outliers, automated processes, or abnormal usage behavior. To assess robustness, we repeated the analysis under alternative percentile cutoffs (e.g., 95th and 99th percentiles). The dominant topics and representative transition paths remained qualitatively consistent across these specifications.

##### Treatment of system-generated and highly frequent actions

Notably, when topic modeling algorithms are used for text-only data, further preprocessing is usually needed, e.g., stemming, lemmatization, and removal of stop words. In our case, such processing is not required since the action descriptions are taken unchanged and are considered words. To remove noise that might affect the quality of the generated topics, we exclude overly frequent actions that provide minimal information about the journeys. An example of such an action is logging into the system. Since this type of action occurs in every session, it is not indicative of a particular type of journey and does not reveal anything interesting about the customer’s intent or the task to be completed.

#### Topic extraction

To obtain the most important journeys from many sessions, one needs to cluster the sessions into a reasonable number of groups that represent the most important journeys. Since we know that theoretically, there can be different efficient clustering algorithms for such a task, we assume that certain algorithms are better suited for our data. In Section “Results on real-world data”, we present implementations of different types of clustering algorithms, some of which have been used in previous works to solve similar problems. As will be shown later, LDA is the most suitable algorithm for this task, so we recommend using it as part of the proposed framework, although a hierarchical version of LDA can be used alternatively.

**Fig. 3:**
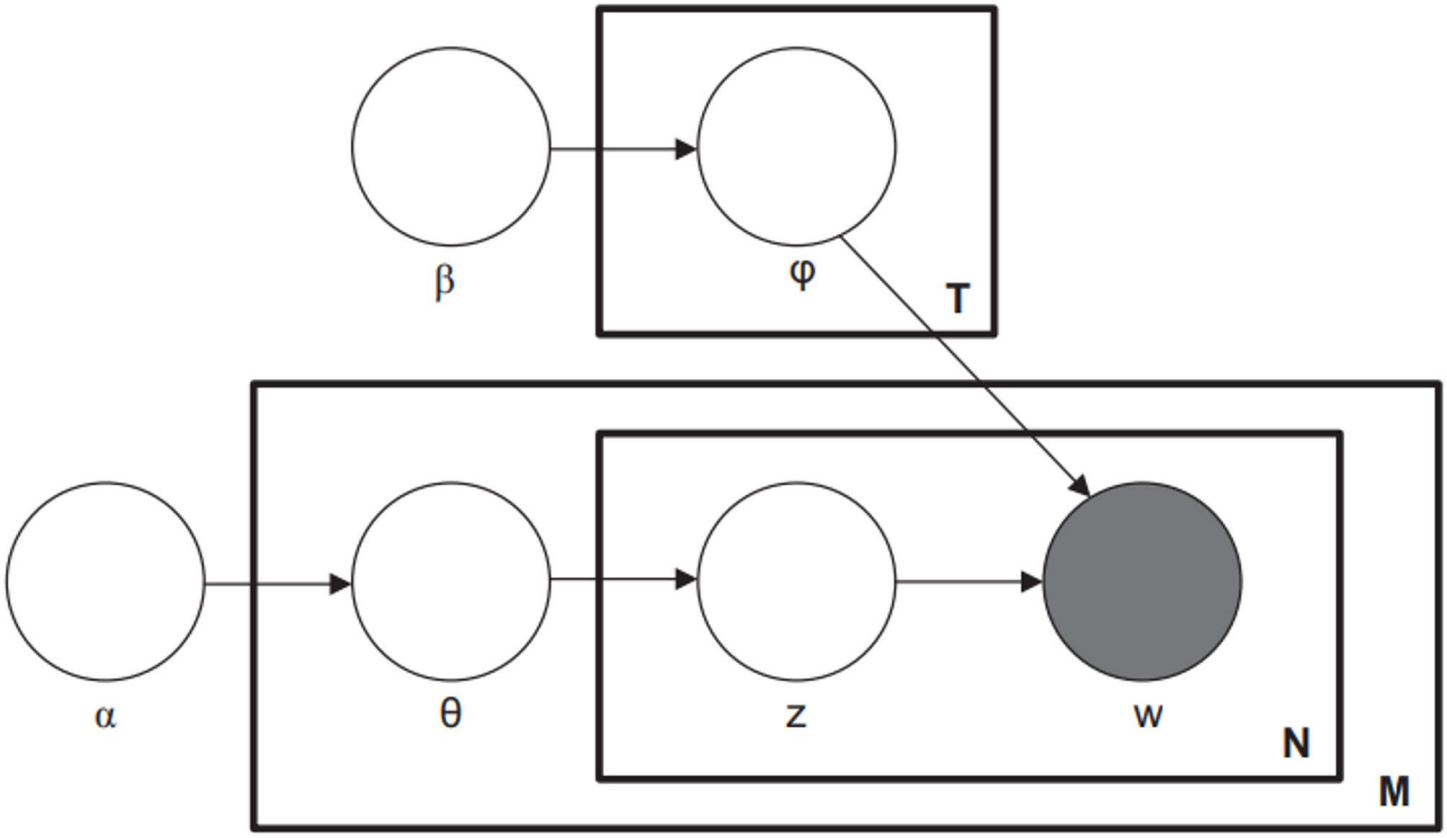
Plate notation representation of the LDA model.

LDA is based on a Bayesian network, as described in Fig. 3.

Each box (plate) represents replicated entities. The outer plate represents documents (M), whereas the inner plate represents the repeated word positions (N) in a given document. Each position is associated with a choice of topic (z) and word (w).

In Fig. 3, $\alpha$ is a parameter of the uniform Dirichlet prior of the per-document topic distributions; $\beta$ is a parameter of the uniform Dirichlet prior of the per-topic word distribution; ${\theta}_{i}$ is the topic distribution for document $i$; ${\phi}_{j}$ is the topic distribution for word $j;{z}_{ij}$ is the topic for the $j$-th word in document $i$; and ${w}_{ij}$ denotes a specific word. ${w}_{ij}$are the only observable variables.

A word $w$ is the basic unit of data, and each document is constructed of words.

In the dataset, there is a set of $D$ documents $\{{d}_{1},{d}_{2}.....{d}_{D}\}$, where each document is viewed as a mixture of topics $z$, and topics are distributed over words (each topic can be represented by the list of words associated with the conditional probability $p\left(w\right|z)$). For each document $i$, the probability of a word ${w}_{ij}$ is given by $p\left({w}_{ij}\right)={\sum}_{t=1}^{K}p\left({w}_{ij}\right|{z}_{ik}\left)p\right({z}_{ik})$, where $K$ is the number of topics. $p\left({w}_{ij}\right|{z}_{ik})$ and $p\left({z}_{ik}\right)$ are assumed to follow Dirichlet distributions with hyperparameters $\alpha$ and $\beta$, respectively.

LDA learns the model hyperparameters during an optimization phase based on EM. In most cases, Gibbs sampling is used for the optimization phase. Once the model is trained, Bayesian deduction enables us to extract the set of topics that describes each document based on $p\left(d\right|z)$.

LDA enables us to infer inherent patterns of digital customer behavior from digital event logs by generating “topics”. Originally, topics represented meaningful probabilistic distributions over words and documents. In our case, they represent meaningful distributions over actions and sessions.

Each action performed by a customer is mapped to a word in the model, and each session (sequence of actions) is mapped to a document. The list of all sessions is mapped to a corpus. Since LDA is an unsupervised algorithm that does not require a priori classes (or topics) or labeled data, it is suitable for our problem.

LDA accepts the following input:

- A list of sessions;
- The number of desired topics ($k$).

**Fig. 4:**
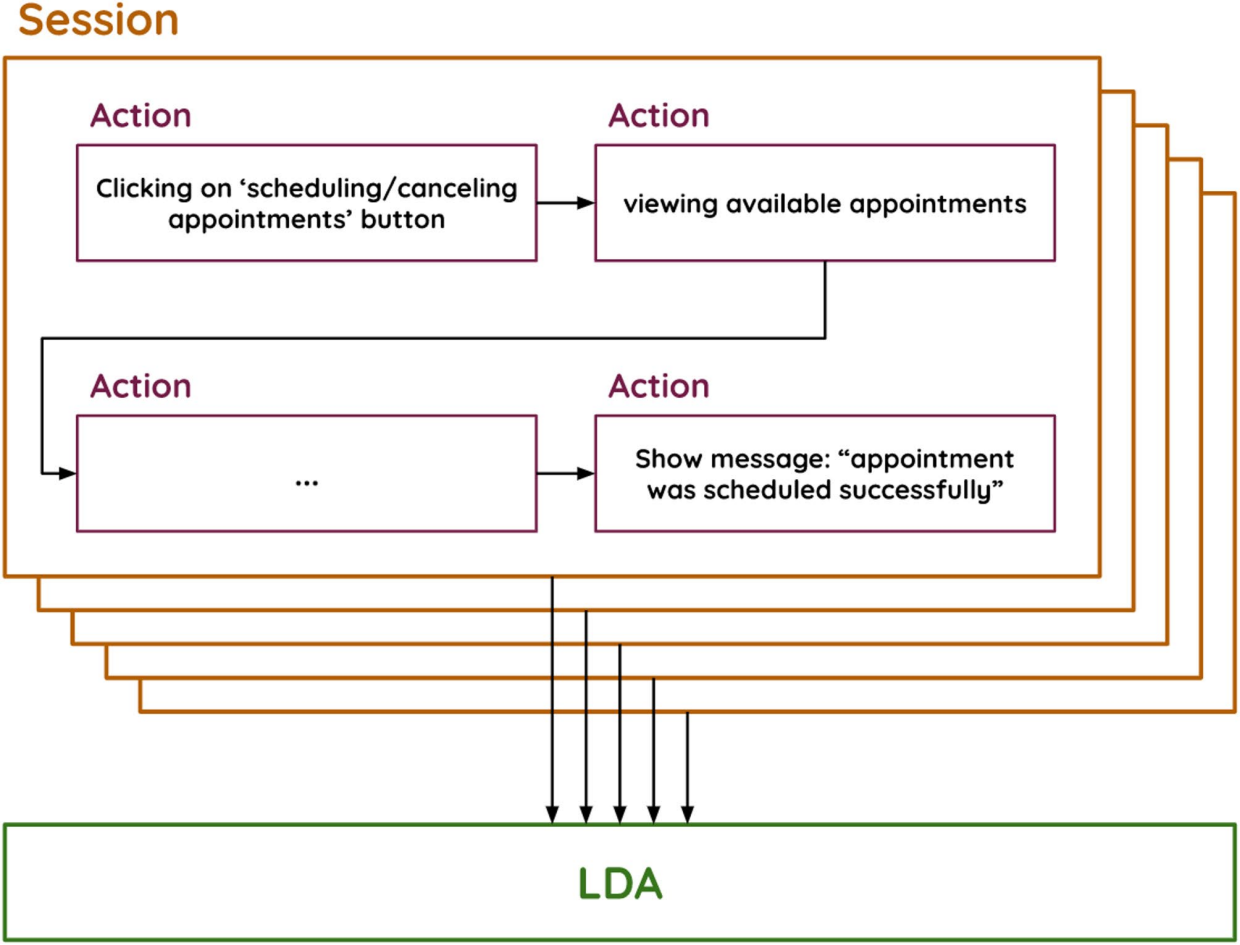
Input values for the LDA model. “Clicking on the ‘scheduling/cancelling appointments’ button” represents an action, and the list of all actions represents a session.

Figure 4 illustrates the data provided as input to the LDA model.

Let $Z=\left\{{z}_{1},{z}_{2},...,{z}_{K}\right\}$ denote the *K* latent topics inferred by the LDA model. Let $S=\{{s}_{1},{s}_{2},...{s}_{N}\}$ be the set of *N* customer sessions provided as an input to the model; and let $A=\{{a}_{1},{a}_{2},...,{a}_{M}\}$ be the set of unique customer actions observed in the data. In LDA each topic ${z}_{k}$ is represented by a multinomial distribution over actions. We denote this topic-action distribution ${p}_{k,m}$, where for any action ${a}_{m}\in A,{p}_{k,m}=\mathrm{P}\mathrm{r}\left({a}_{m}\right|{z}_{k})$ is the probability of observing action ${a}_{m}$ conditional on topic ${z}_{k}$.

$${z}_{k}=\left[{p}_{k,1},...,{p}_{k,M}\right];{p}_{k,m}:=\mathrm{P}\mathrm{r}\left({a}_{m}\right|{z}_{k}),\forall k\in[1,\dots,K],m\in[1,\dots,M]$$

The higher the probability is, the more representative the action is of the topic. All action probabilities for a single topic sum to 1: ${\sum}_{m=1}^{M}{p}_{k,m}=1,\forall k\in[1,\dots,K]$. In addition, LDA returns for each session a topic-mixture vector ${s}_{n}$ whose components ${p}_{n,k}=\mathrm{P}\mathrm{r}\left({z}_{k}\right|{s}_{n}),$ represent the posterior probability that session ${s}_{n}$ is associated with topic ${z}_{k}$. ${s}_{n}=\left[{p}_{n,1},...,{p}_{n,K}\right];{p}_{n,k}=\mathrm{Pr}\left({z}_{k}|{s}_{n}\right),\forall k\in[1,\dots,K],n\in[1,\dots,N]$. The higher the probability is, the more representative the topic is of the session. These probabilities also sum to 1: ${\sum}_{k=1}^{K}{p}_{n,k}$=1 $\forall n\in[1,\dots,N]$.

**Fig. 5:**
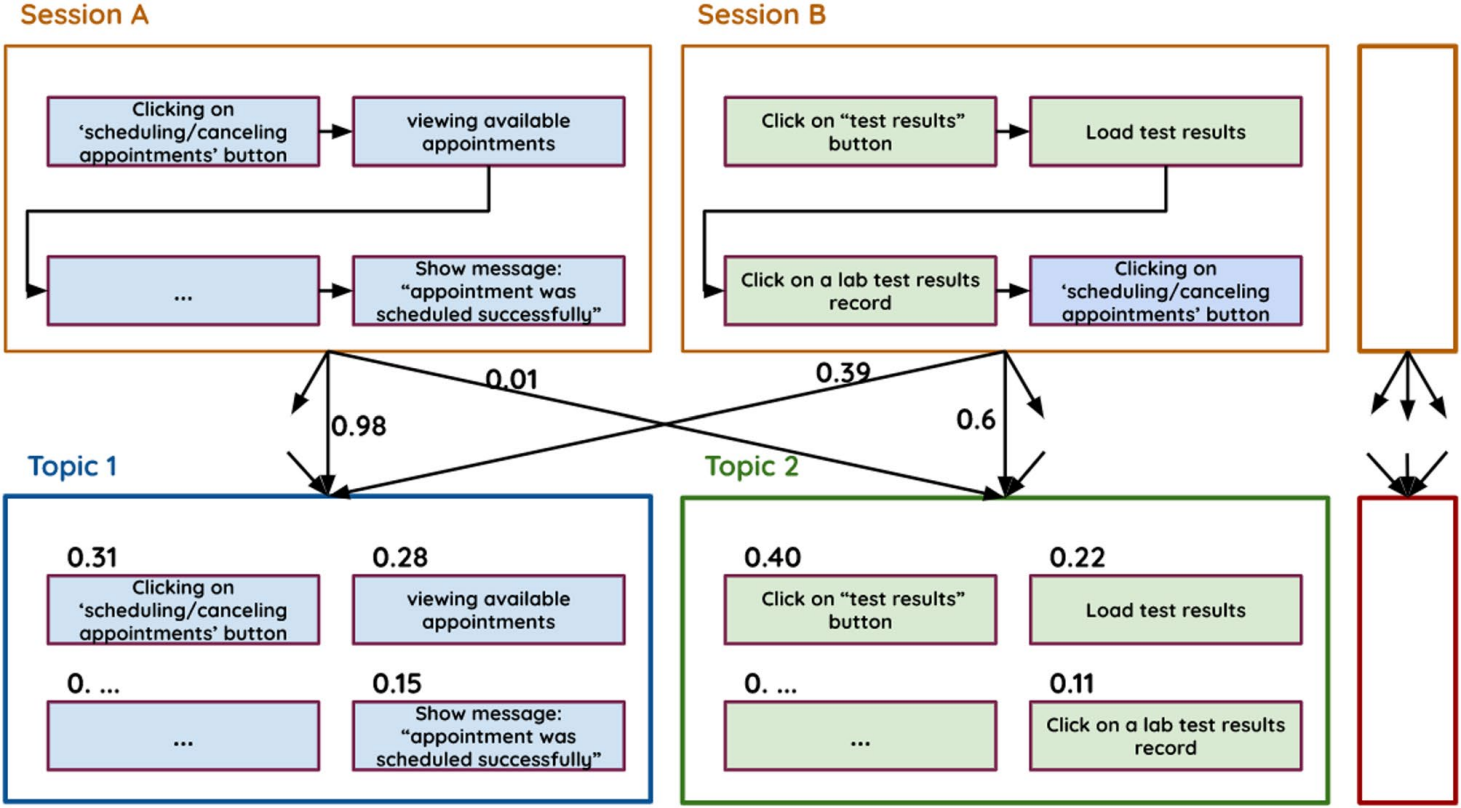
Input–output example of the LDA model.

Figure 5 shows the outputs of the LDA model.

Figure 5 shows examples of the model’s inputs, namely, Session A and Session B, along with a possible long list of other sessions. It also shows examples of the outputs of the model, namely, Topic 1 and Topic 2, with the possibility of additional topics. Each topic consists of a list of actions associated with a probability of belonging to that topic. These actions are sorted by their probabilities in descending order so that the most representative actions can easily be identified and the conceptual idea behind each topic can be understood. For example, Topic 1 appears to be related to the journeys of scheduling a doctor’s appointment, whereas Topic 2 is related to viewing laboratory test results. The ease with which the meaning of each topic is perceived stems from our ability to interpret, which plays an important role in ensuring that our results make sense.

Another output of the model is the probability assigned to each session per topic. For example, Session A is assigned a higher probability of belonging to Topic 1 (0.98) than to Topic 2. The representative actions indicate that this session involves making a doctor’s appointment, which reflects the meaning of Topic 1. Session B, on the other hand, contains actions related to both laboratory test results and scheduling a doctor’s appointment. Therefore, LDA splits the probabilities more evenly between the two topics so that the probabilities of being assigned to Topic 1 and Topic 2 are 0.39 and 0.6, respectively.

#### Finding the best number of topics

One of the major challenges in unsupervised learning tasks is evaluating the performance of the model. Since the number of main journeys is not given beforehand, one goal is to select the best number of topics based on our data. In general, we consider two approaches to achieve this goal: qualitative and quantitative. The first method requires looking at the observed topics and using human judgment and domain expertise to ensure that the obtained topics are meaningful. However, since the goal is a generic method with minimal manual effort, we must evaluate the proposed method systematically via quantitative measures. In the absence of ground-truth information, internal validity indices are most appropriate metrics because they evaluate performance based on the distribution of entities within clusters (internal indices) rather than the distribution of labels across clusters (external validity indices). Therefore, we use the coherence metric for evaluation, as discussed later.

#### Hierarchical latent Dirichlet allocation extension

An alternative to using LDA is to use a hierarchical version of LDA, termed hLDA. We show in a later section that hLDA performs well on our data. The use of hLDA has two main advantages:

1. It supports a hierarchical dimensionality of the topics, which enables customer journeys to be broken down at different levels of granularity.
2. There is no need to specify the number of topics as an input. hLDA automatically learns and optimizes the best number of topics based on the given data.

The leaves of the tree can be used to convert hierarchical topics into flat topics.

Although hLDA can be unstable when used for text data and requires considerable effort in tuning its hyperparameters, as mentioned in Lindgren^44^, we found it useful for our problem; this could be related to the fact that digital customer journeys are less variable than are documents because of the schematic structure and constraints in the digital system.

In the next section, we apply hLDA to a real-world dataset.

### Cluster profiling

#### Cluster preprocessing

In this section, we aim to build a per-topic Markov model. However, since topics can be considered soft clusters, we first adjust the results so that each session is assigned to only one topic. To do this, we define the “dominant topic” per session, i.e., the topic with the highest probability according to LDA:

${D}_{topic}\left({s}_{i}\right)=argmax\left(LDA\right({s}_{i}\left)\right)\forall i=\mathrm{1,2}....n$, where ${s}_{i}$ is session $i$, where $n$ is the total number of sessions, and where $LDA\left({s}_{i}\right)$ is a vector of size $k$ returned by LDA that contains the probabilities of session $i$ belonging to each of the $k$ topics.

The conversion of topics to hard clusters has various drawbacks related to the loss of information; however, it is still simple and intuitive, requires only a small number of computations, and provides an immediate understanding of the obtained clusters. Note that this step is optional, as Markov models can be created without it (e.g., by creating weighted Markov models based on the probabilities with which sessions are assigned to each topic).

#### Cluster profiling using Markov models

Each cluster can be described by a conditional distribution function of actions depending on previous actions. A Markov model is used to model each cluster to capture the sequential dependencies between actions. A simple first-order Markov model with relatively low computational complexity can be used to highlight repeating patterns in a customer’s behavior.

In general, the possible actions in a digital system form a state space $\{{s}_{1,}{s}_{2,}....{s}_{n}\}$. A Markov chain holds the likelihood of transitioning from state ${s}_{i}$ to state ${s}_{j}$. The probabilities for all transitions are presented in a transition probability matrix ${\left[P{r}_{ij}\right]}_{nXn},$ where ${\sum}_{j=1}^{n}{p}_{ij}=1$ for every $i$.

We let ${A}_{t}\in\left\{{a}_{t}\right\}$ be a random variable representing an action performed in step $t$. The conditional probability of the action performed in step $t+1$, accounting for previous actions in this case, is then approximated via a first-order Markov chain (a transition matrix), namely, $P({A}_{t}+1=x|{A}_{1}={a}_{1},{A}_{2}={a}_{2},....{A}_{t}={a}_{t})\approx P({A}_{t}+1=a|{A}_{t}={a}_{t})$. Note that the set of actions is finite. The size of the transition matrix corresponds to the number of unique actions in the sessions associated with the cluster.

**Fig. 6:**
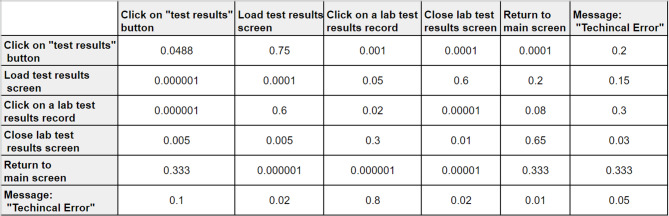
An example of a transition matrix.

Figure 6 shows an example of a transition matrix. The columns of the matrix represent the actions that the customer performed in step $t+1$, whereas the rows represent the action that the customer performed in step $t$. The entries of the matrix are the conditional probabilities $Pr({A}_{t}+1={a}_{t+1}|{A}_{t}={a}_{t})$. The main drawback of a first-order Markov model is that it does not capture complex sequential patterns; therefore, we will show later how this can be approached via a higher-order Markov model.

By representing each cluster as a first- or higher-order Markov chain, one can gain meaningful insights into sequential customer behavior, as explained next.

An alternative to using a first-order Markov model is to use a higher-order Markov model. In an nth-order Markov model, the probability of a state depends on the probability of the *n* previous states such that

$$P\left({x}_{i}\right|{x}_{i-1},{x}_{i-2},....{x}_{1})=P({x}_{i}|{x}_{i-1},....{x}_{i-n})$$

Although higher-order Markov models can capture more information and memory than can first-order Markov models, they have several disadvantages: they are generally computationally intensive and require a greater number of model parameters to express all possible states. Indeed, as the order of the model increases, the number of parameters grows exponentially.

### Cluster analysis

#### Finding the most representative paths

In this part, we show how to extract the most representative paths in each cluster via the first-order Markov model presented above. With the ability to automatically generate the “flow” of actions performed by customers, one can detect gaps between actual and “expected” or planned behavior.

The most representative paths are identified using a *maximum log-likelihood criterion* under the cluster-specific Markov model. Particularly, paths with the highest likelihood are interpreted as those best capturing the typical transition dynamics within the cluster, providing a statistically grounded measure of representativeness rather than a heuristic selection. Since event-log data are collected in distinct time periods, representative paths may evolve over time. This temporal variability allows the framework to capture shifting behavioral patterns, thus it ensures that diverse usage scenarios are systematically analyzed.

We let $\left\{{A}_{i}\right\}i=1,\dots T$ be a path of actions in the Markov chain, and we let ${P}_{\theta}({A}_{1},...{A}_{T})$ be the probability of observing the path when $\theta$ is the probability parameter value (the likelihood function for $\theta$). Using the definition of conditional probability, one can define

$${P}_{\theta}({A}_{1},....{A}_{T})={P}_{\theta}\left({A}_{T}\right|{A}_{T-1},....{A}_{1})\cdot{P}_{\theta}({A}_{1},....{A}_{T-1})$$

Following the Markovian property, one knows that ${P}_{\theta}\left({A}_{T}\right|{A}_{T-1}.....{A}_{1})={P}_{\theta}({A}_{T}\left|{A}_{T-1}\right)$, which simplifies the equation to ${P}_{\theta}({A}_{1},....{A}_{T})={P}_{\theta}\left({A}_{T}\right|{A}_{T-1})\cdot{P}_{\theta}({A}_{1},....{A}_{T-1})$. For $T$ steps, one obtains ${P}_{\theta}({A}_{1},....{A}_{T})={\prod}_{i=1}^{T}{P}_{\theta}\left({A}_{i}\right|{A}_{i-1})$, where ${A}_{0}$ is the initial state of the process. As the likelihood becomes very small, it is much more convenient to use the logarithm of the likelihood. Thus, instead of multiplying the likelihoods, we sum the log-likelihoods in the following manner: $L\left(\theta\right)={\sum}_{i=1}^{T}log\left({P}_{\theta}\left({A}_{i}\right|{A}_{i-1})\right)$. Accordingly, for every session, we calculate the log-likelihood based on the likelihood function (i.e., the transition matrix) of the cluster to which it belongs. The session with the maximum log-likelihood reflects the most likely session and is selected as the representative session.

Notably, the size of the session (the number of actions performed) strongly influences the log-likelihood value. In other words, longer sessions naturally have lower log-likelihood values than shorter sessions. To prevent bias toward shorter sessions^45^, we set a lower bound on the session length, which is the median session length. Although this approach may result in longer or shorter representative sessions than expected, the results are reasonable and practical. However, in future research, we recommend finding the optimal session length for the most representative sessions or applying a normalization procedure to enable a better comparison among paths of various lengths.

*Design-intent baselines and deviation scoring (optional step).* In many digital services, product requirements and UX specifications implicitly define an intended “planned path” (e.g., $A\to B\to C$). While our framework is data-driven and does not require such specifications, it can incorporate *design intent* as an additional evaluation lens for actionability: for a given task, we define a set of intended paths (or an intended transition graph) derived from product specifications, click-flow diagrams, or QA scripts, and we quantify *deviations* of observed sessions from this baseline. Thus, for each observed session $s=({A}_{1},\dots,{A}_{T})$, we compute a conformance score using one (or more) of the following:

(i) *Log-likelihood ratio* between a “design” Markov chain and the cluster-specific Markov chain (transition-level conformance); (ii) Missing/Skipped/Swapped step counts (interpretable deviation types, e.g., $A\to C\to B$ instead of $A\to B\to C$).*These deviation scores* can be aggregated per topic/cluster to identify where the designed journey is systematically violated (suggesting design friction, unmet user needs, or technical failures) and to prioritize actionable redesign opportunities.

#### Detecting leaks between clusters

In this part, we look for irregular sessions that reflect unexpected behavior; for example, sessions in which customers perform two tasks in a row, such as viewing their laboratory test results and making an appointment with a doctor, could be detected by the proposed method and enable us to identify interesting behaviors based on the data. To find such sessions, one can look for inconsistencies in the assignment of sessions to (hard) clusters. As shown earlier, LDA outputs a probability for each session to be assigned to each of the *K* topics, and the sum of these probabilities across all clusters is 1. Hard clustering with LDA is performed by assigning each session to the cluster with the highest LDA probability. With respect to the Markov model, hard clustering is performed by computing the log-likelihood of each session based on the transition matrices across all clusters. We then assign each session to the cluster that obtains the maximum log-likelihood score. After performing these two hard clustering processes, each session has two clusters to which it is assigned: one based on LDA and the other based on the Markov models. Next, we look for sessions with a mismatch between these clusters and try to determine which clusters there are leaks. We propose a simple method of applying the Pareto principle to the LDA probabilities. In particular, each session has k values for the LDA probabilities—one per cluster. These probabilities can be sorted in descending order for each session. By taking a cumulative sum of these probabilities and considering the topics for which this sum falls below 0.8, we obtain the topics with leaks. To exclude negligible or irrelevant topics, we remove topics with a low LDA probability of less than 1/*K* (where *K* is the number of clusters).

#### Detecting actions that lead to undesirable outcomes

The Markov models described earlier can also be used to detect actions that have a high probability of producing undesirable results, such as triggering automatic errors or churn of customers in the middle of their journeys without completing their requested tasks. By using first- (or higher-) order Markov models, one can identify actions or sequences of actions that are most likely to be followed by such undesirable actions. An example of this is identifying which actions will cause a session to terminate; this helps identify actions that unexpectedly lead to churn when they should not. In the next section, we present an example of such a real-world scenario.

### Post cluster analysis

In this part, the proposed method analyzes how different customer characteristics (e.g., age, gender, geographic location, device used, and general health status) can affect session characteristics and customer journeys. For example, one can determine whether the most representative sessions differ among age groups or if one can detect undesirable occurrences during journeys on certain types of phone devices.

Furthermore, this part aims to evaluate the main journeys in terms of various performance metrics, e.g., session length and number of loops/errors during the journey, to further optimize them for different segments of customers. One example is to design different sessions for specific tasks that omit actions that were found to be less relevant for some customer segments (e.g., young customers in a healthy state do not need to open the same windows as older customers do).

## Results on real-world data

In this section, we present an implementation of the proposed framework using a real dataset.

### Data

Our dataset contains event-log data that were collected from a mobile application provided by *Maccabi Healthcare Services*, a large health maintenance organization (HMO) in Israel. The event logs record customer activities and interactions within the application. Each log contains a timestamp, an action description, a customer identifier and a session identifier that helps track the customer’s navigation path. Each session begins when the customer logs into the application and ends when the customer logs out or when the customer is inactive for a few minutes. The events can be manual actions (e.g., clicking a particular key) or actions automatically generated by the system (e.g., loading a screen). In this work, we do not distinguish between these two types of actions.

There are approximately 120,000 average daily active customers in the application, generating almost 5.5 million events that can be grouped into approximately 350,000 sessions. Each session contains 15 actions, on average. For this research, we used event log activities of 10,000 randomly selected customers over a period of six months between July 2020 and December 2020. In total, approximately 7.5 million events are represented by 800 unique actions and are grouped into approximately 380 K sessions. Finally, for simplicity, we translated the action descriptions from Hebrew to English via Google Translate. We anonymized all the data and results according to privacy guidelines.

The first step of data preprocessing was to group individual event log records into session records. Each session record contains a list of action descriptions, sorted by time of occurrence. Our data also include session details (e.g., device type and system version) and customer demographic information (e.g., age, gender, and region). In the second step, we cleaned the data to remove noisy and extreme observations. Thus, we excluded very short sessions with fewer than 3 actions and long sessions with more than 200 actions.

### Discovering the main journeys via real-world data

#### Benchmark: session clustering & evaluation methods

To decide which algorithm should be used to discover the main journeys, we compared various algorithms, some of which were used for similar tasks in previous works. To do so, we conducted a series of experiments and compared several clustering algorithms via two evaluation metrics, *coherence* and the *Calinski–Harabasz index*, where we chose the number of clusters in increments of 2 between 2 and 42 (the upper bound was set to 42 based on information provided by domain experts).

We considered six algorithms, some of which have previously been used to solve similar problems. These algorithms differ in nature (e.g., soft vs hard clustering), and some are not exactly clustering algorithms but topic models adapted to cluster sessions:

1. Latent Dirichlet al.location (LDA) is the algorithm in our proposed framework. It is based on the method presented in Campbell et al.^33^.
2. Hierarchical latent Dirichlet al.location (hLDA) is an extension of the LDA model. As presented in Blei et al.^37^, hLDA is a nonparametric model that generates each session as a superposition of topics.
3. Mixture of *K* Markov models: In this process, we follow^24^, which presents an algorithm for customer journey mapping. We assume that each session is a multidimensional point in space that is generated from a mixture of *K* Markov models. To find the *K* clusters, we apply an EM algorithm.
4. K-means is based on doc2vec embedding over a Euclidean distance metric^14^. This algorithm has proven successful in categorizing documents and is fast and simple. Although it has not been used for web sessions before, we decided to test it on our data because of its past success and simplicity.
5. K-means & cosine similarity of term frequency vectors: In this procedure, we follow^15^ by converting each session (sequence of actions) into a term frequency vector and applying the *K*-means algorithm to the pairwise cosine similarity matrix of the term frequency vectors.
6. Hierarchical clustering based on shingles (CJM-Ex). In this method, we follow^17^ and compute a pairwise distance matrix between sessions based on the shingle distance metric. Then, we apply hierarchical clustering to the distance matrix.

It is important to mention a considerable drawback of the last algorithm, namely, hierarchical clustering based on shingles, which has a memory complexity limitation. This limitation stems from the need to create a similarity matrix of size *N*×*N*, where *N* is the number of sessions, which can grow very large. Since our computer resources are not capable of manipulating such a similarity matrix for the entire dataset (nearly 350,000 sessions), we excluded it from this experiment.

### Evaluation methods

Since the above algorithms are a mixture of soft (e.g., LDA) and hard (e.g., K-means) clustering approaches, as well as unsupervised approaches that do not rely on ground-truth journey labels, we use evaluation metrics that are well-suited for these types of algorithms. In particular, we used two intrinsic evaluation metrics, namely, the Calinski–Harabasz index^46^ and topic coherence^47^. The Calinski–Harabasz index is a popular measure for evaluating classical clustering algorithms and is expressed as the ratio of the variance between the clusters and the total variance within the clusters. Topic Coherence is a popular metric for topic modeling algorithms that was found to be highly correlated with human interpretability^48,49^. There are various variations of topic coherence metrics, and in this research, we specifically use the one proposed by Roder et al.^47^, that was shown to outperform other coherence metrics, such as NPMI^50^ and UMASS^51^.

As discussed, for each cluster, representative paths are selected via a maximum log-likelihood criterion under the cluster-specific Markov chain. To mitigate the bias toward shorter sequences, we restrict candidates using a minimum length threshold (set to the median session length within the cluster).

Finally, in some customer journey settings, exhaustive labels for “correct” journeys are available. In such cases, the discovered clusters and representative paths can be validated against the labeled “expected” journeys using standard external validation measures (e.g., agreement/accuracy at the cluster level, and edit-distance or conformance scores at the path level). In the more common case where exhaustive labels are not available, one can use design-intent baselines when available (planned vs observed flows, as discussed in Secion “Evaluation methods” below). Finally, one can also use domain-expert review of topic interpretability and actionability. Where additional external signals exist (e.g., satisfaction surveys, support tickets, or A/B test outcomes), they can be used to further validate and quantify the downstream impact of detected friction points.

#### Results

**Fig. 7:**
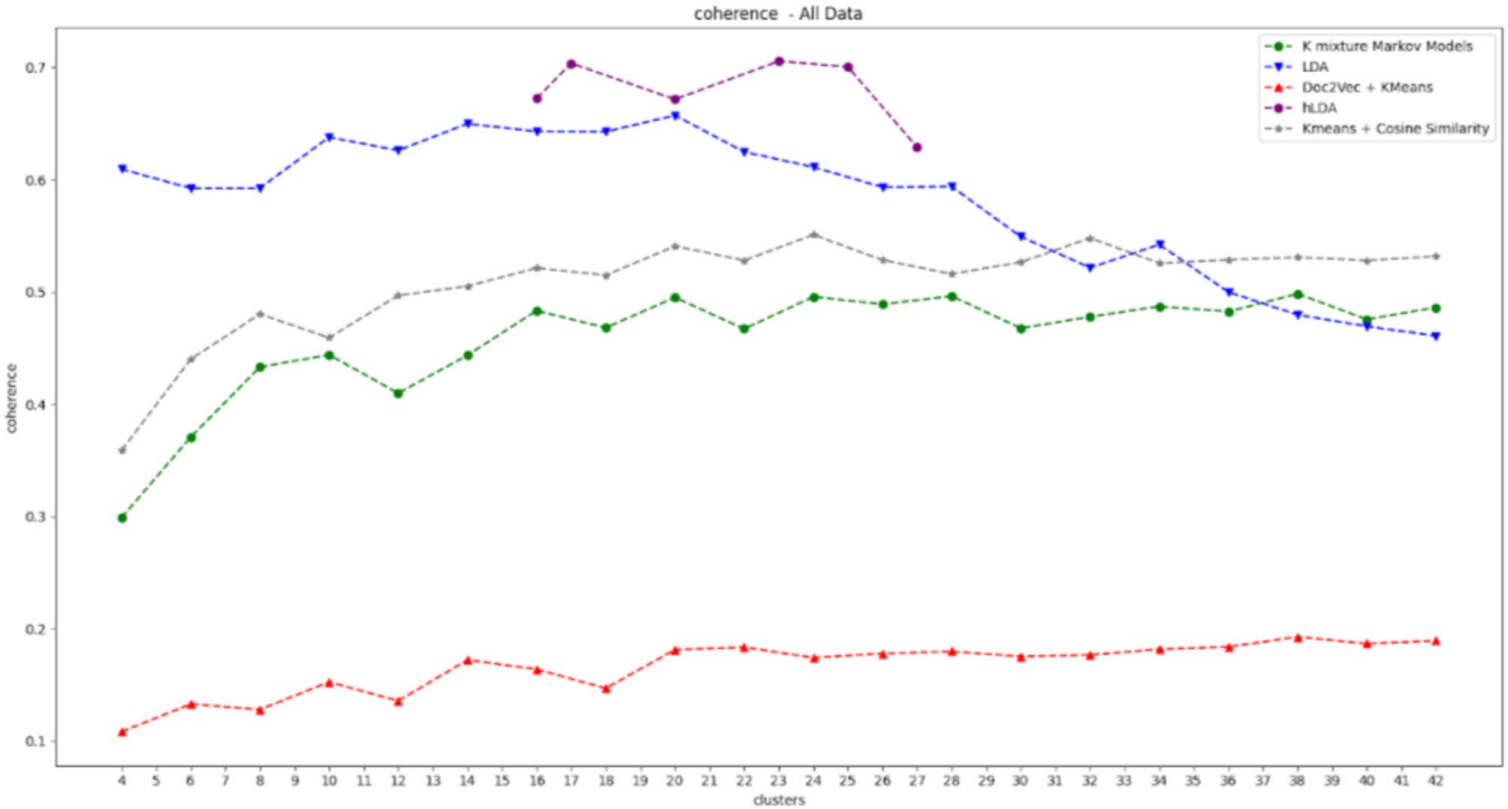
Coherence of the five clustering procedures. The x-axis represents the number of clusters, and the y-axis represents the coherence.

**Fig. 8:**
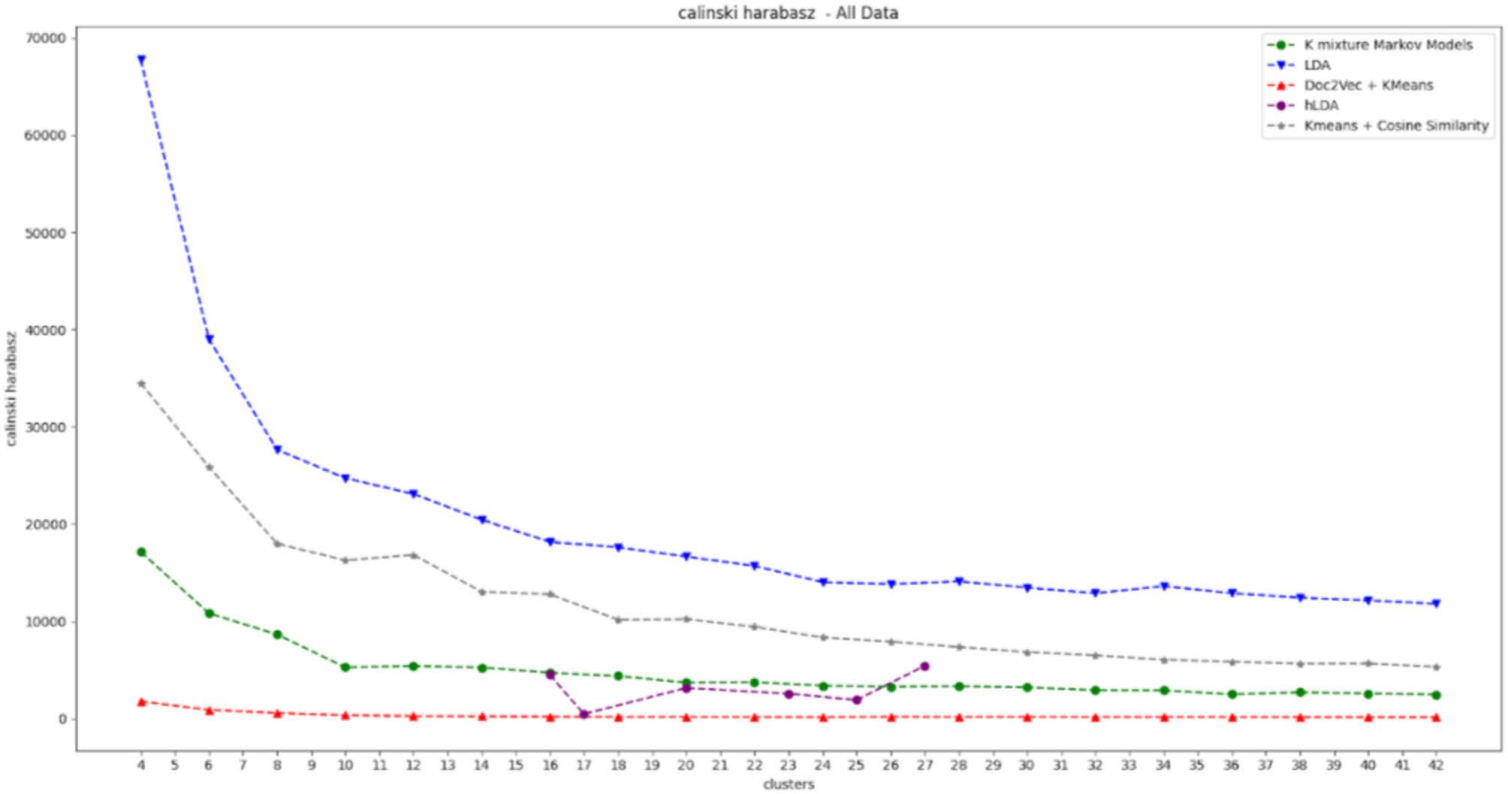
Calinski-Harabasz index values of the five clustering procedures. The x-axis represents the number of clusters, and the y-axis represents the Calinski–Harabasz index.

Figure 7 shows the coherence values, while Fig. 8 shows the Calinski–Harabasz values over a range of numbers of clusters.

Since hLDA automatically learns the number of topics/clusters, it has some missing numbers of clusters.

As shown in the above graphs, LDA is the best algorithm in terms of the Calinski–Harabasz index and obtains the second-best coherence for the majority of clusters. This implies that it is capable of creating clusters that are both homogeneous and well separable.

Although hLDA has the best coherence values (for a subset of clusters), this is not the case for the CH index.

The K-means algorithm, which is based on TF-IDF and cosine similarity, has an intersection with LDA in the coherence graph but has lower coherence for most of the clusters and lower Calinski–Harabasz values for all the clusters. The algorithm with the worst results for both coherence and CH index is the K-means algorithm, which is based on the doc2vec procedure.

In summary, LDA achieves the best balance between the two performance metrics and therefore was chosen for implementation in the proposed framework.

### Implementation of LDA to find the main journeys

**Fig. 9:**
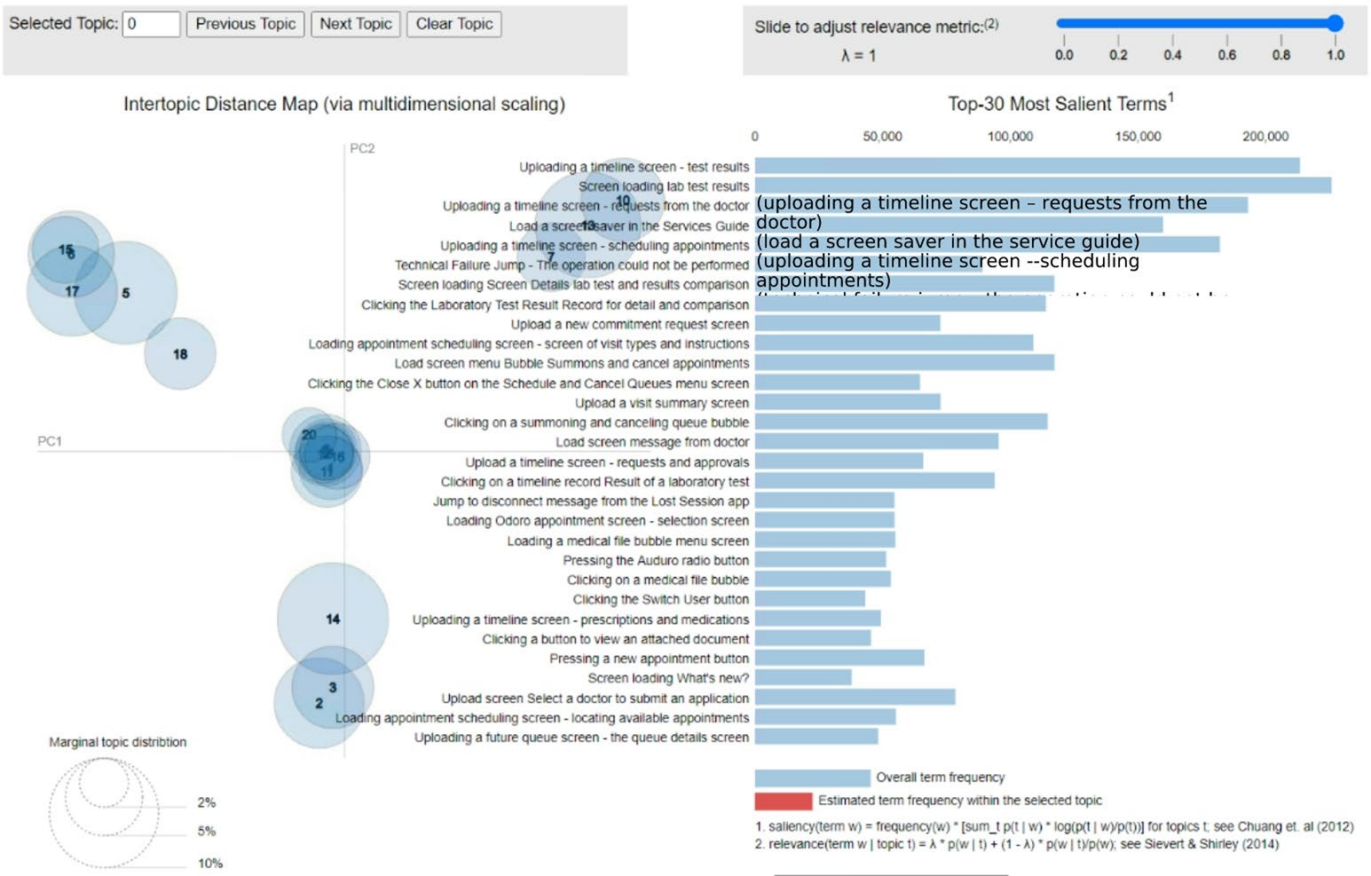
Overview of the obtained LDA topics.

Based on the above-described benchmark and experiments, we observed that topic extraction and session clustering on the analyzed data are best performed via LDA. Therefore, we applied LDA with 20 topics to the data since this number of topics resulted in the highest coherence. Figure 9 illustrates the obtained topics on a two-dimensional axis via pyLDAvis^52^.

The topics are plotted in a two-dimensional plane as circles whose centers are determined by computing the distances between topics and then using multidimensional scaling to project the intertopic distances onto two dimensions, as described in Sievert and Shirley^52^. The size of each circle reflects the number of sessions corresponding to that topic, which indicates which journeys are frequent and which are rare. The blue bars represent the top 30 actions with the highest saliency (overall frequency in the dataset).

**Fig. 10:**
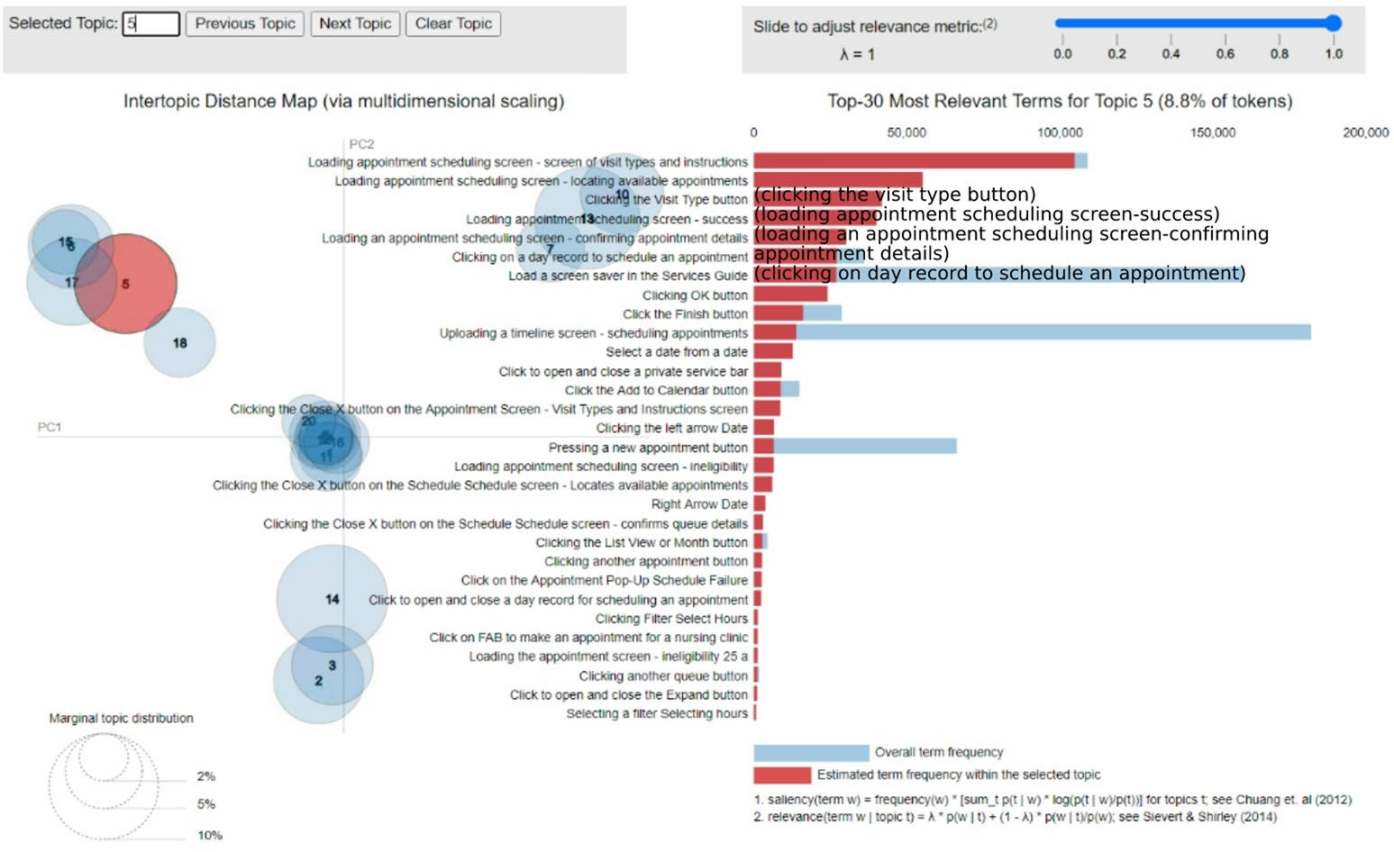
Representation of a specific LDA topic.

Figure 10 shows how it is possible to drill down to a specific topic and understand its focus and meaning.

The interactive graph enables one to hover over a specific circle (topic) and obtain the most significant actions of the topic. The red bars represent the estimated action frequency within the topic, whereas the blue bars represent the overall action frequency (over the whole dataset).

#### Example of an obtained topic

Figure 10 illustrates a specific topic (Topic #5 in this example). Hovering the mouse cursor over the corresponding circle shows the most frequent actions related to scheduling a doctor’s appointment:

- “Loading appointment scheduling screen—screen of visit types and instructions”.
- “Loading appointment scheduling screen—locating available appointments”.
- “Clicking the visit type button”.
- “Loading appointment scheduling screen—success”.
- “Loading appointment scheduling screen—confirming appointment details”.

From the gaps between the blue and red bars, one can tell which actions belong mainly to this topic and which belong to multiple topics. For example, the action “Loading the scheduling screen—Success” (the fourth action from the top) seems to occur only in this topic. In contrast, the action “Load a screen saver in the Services Guide” (7th from the top) also seems to occur in other topics, which makes sense since this action acts as a “hub” for multiple actions that a customer can take. One can also use domain experts’ judgment to decide whether the generated clusters are reasonable and coherent, as in this study when the results were presented to experts. In this case, cluster 5 appears to be quite coherent: the main actions in this topic are all related to scheduling. An indication of an incoherent topic would be a mixture of “unrelated” actions, such as sending requests to a doctor along with viewing a laboratory test result and scheduling a doctor’s appointment.

### Implementation of hLDA to find the main journeys

**Fig. 11:**
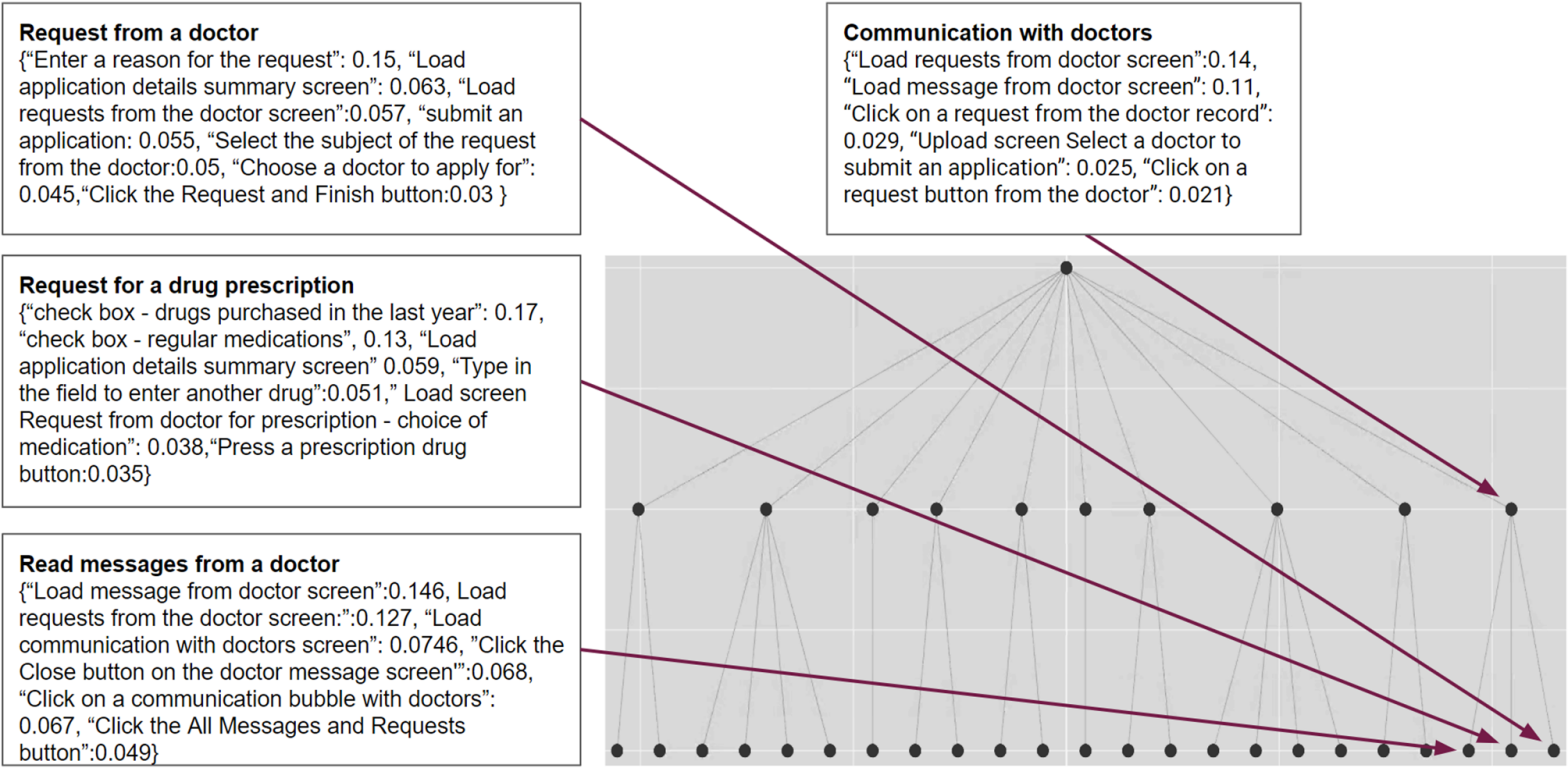
Visualization of the output of hLDA.

Although LDA outperformed the other methods, hLDA also produced good results. The strength of hLDA lies in its ability to break down the output into journeys at different levels of granularity, which is not possible with conventional LDA. In this section, we present an implementation of hLDA using the same data and present the results in Fig. 11. The output is a tree in which each node represents a topic, and the level of the tree indicates the abstraction level of the topic. Thus, the root of the tree represents all available sessions in the data. For example, the rightmost branch of the tree is a hierarchical arrangement of topics related to journeys where customers interact with their doctors. The node at the first level of the tree is a summary of all journeys related to such interactions. This node has 3 leaves—each representing a different journey; the first (from the right) is related to submitting a request to a doctor, the second is related to requesting a drug prescription (this requires a separate path to specify the exact drug), and the third is related to reading messages from the doctor. In addition, being able to break down journeys at different levels of granularity also provides the ability to find anomalies in certain types of journeys. If we had more levels in the tree structure, we might be able to distinguish between journeys that were successfully completed and those that resulted in failure. This information would be expressed through specific topics and pave the way to easily finding opportunities for improvement with minimal manual intervention.

### Cluster analysis results

In this section, we follow the cluster analysis phase, as described in Section "Proposed approach: digital customer journey analysis framework", and present two examples of implementation using the data at hand: finding representative paths within clusters and finding actions with high chances of leading to undesirable outcomes.

#### Finding representative paths

As a first step, we find the most representative/probable paths (sessions) in each cluster. In this way, we can detect gaps between the planned paths, i.e., those designed and supported in the application architecture, and the actual paths, i.e., those extracted from the data logs. In many cases, application designers cannot foresee exactly how customers will manipulate the app.

**Fig. 12:**
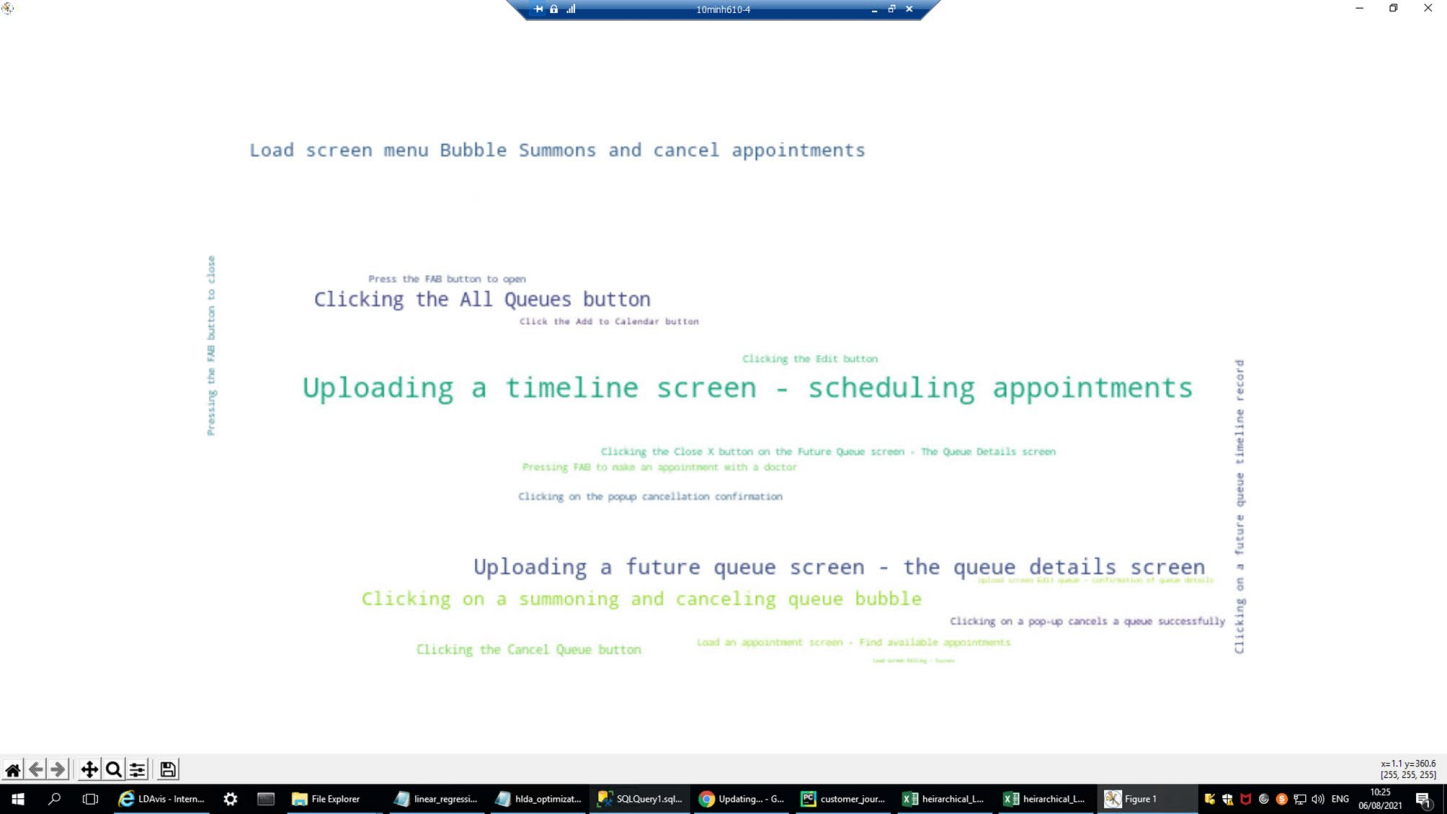
Word (action) cloud of Topic 16.

An example of a cluster where actual journeys differ from planned journeys is cluster 16. Figure 12 illustrates the most common actions in the sessions that were assigned to this cluster via a word cloud. Each word (e.g., “Uploading a timeline screen—scheduling appointments”) represents a single action in the mobile application, and viewing the most important actions in a single graph makes it easy to interpret the meaning of the cluster, i.e., what types of journeys are typical for this cluster. In this case, it is clear from the words (actions) that the journeys are related to scheduling appointments, and there are indications that the focus is on future/existing appointments (e.g., “uploading a *future* queue screen—the queue details” screen). Note that such a comparison between the actual journey and the planned journey can be operationalized via the deviation scores described in Section  “Cluster analysis” (e.g., skipped steps and transition-level conformance), enabling systematic monitoring of design-intent violations across clusters rather than relying on ad-hoc inspection.

**Fig. 13:**
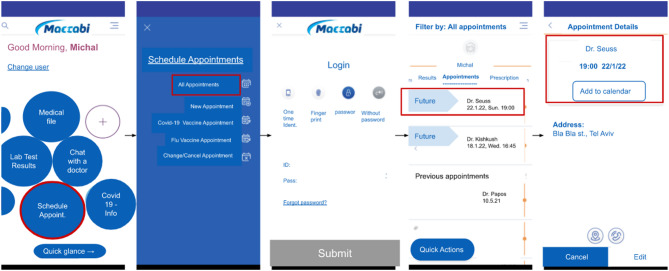
Illustration of the most representative journey of Topic 16.

Figure 13 illustrates the by-design journey as it plays out in the mobile application.

**Fig. 14:**
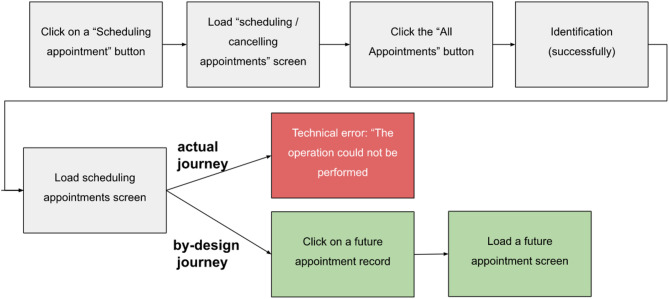
Cluster 16: The by-design journey versus the most likely journey.

Figure 14 shows the planned journey and one of the frequent actual journeys in this cluster. If we compare the planned journey with the most likely journey in this cluster, we notice a gap in the last 2 steps of these sessions. In the planned journey, the customer clicks on a future appointment record (to view its details), and the screen loads. In contrast, in one of the most likely journeys, these last steps are missing, and instead, a technical error is displayed with a message that the operation could not be executed. This insight may be of interest to product designers and developers, who are responsible for providing the best customer experience^5^.

**Fig. 15:**
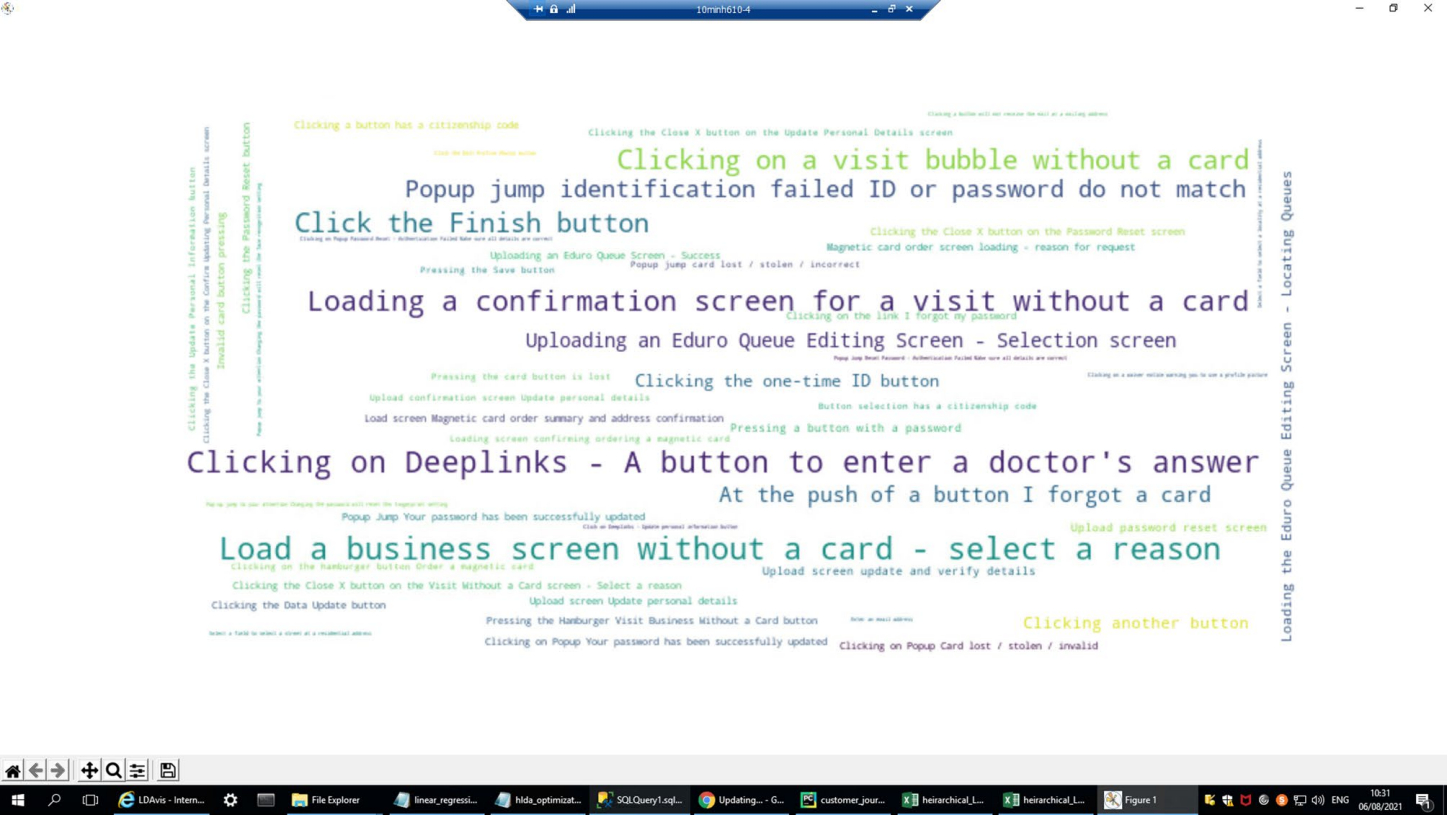
Cluster 19: Word (action) cloud.

In Fig. 15 we present an example of a representative journey that proceeds exactly as planned in cluster 19. Figure 15 shows the word (action) cloud of that topic.

Based on the word cloud, we can see that the journeys in this cluster involve visiting a doctor without a card. This journey is relevant for people who visit a doctor without having their health insurance card with them (because they either forgot or lost it or because it was stolen). In these cases, they must obtain authorization and provide the reason for not having the card to see the doctor.

**Fig. 16:**
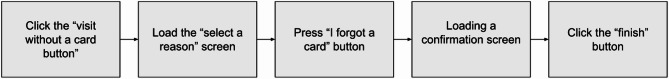
The most representative session of cluster 19.

The representative session of this topic (the one with the highest log-likelihood) is shown in Fig. 16.

This is a fairly simple and short session that plays out exactly as expected, as shown in Fig. 16, which illustrates this journey in the mobile application. Furthermore, since this is the only topic related to a doctor visit without a card, the most likely reason for a doctor visit without a card is that the patient has forgotten the card. One possible approach to improving the customer experience could be sending patients a reminder shortly before the appointment to bring their card.

#### Actions that lead to undesirable outcomes

**Fig. 17:**
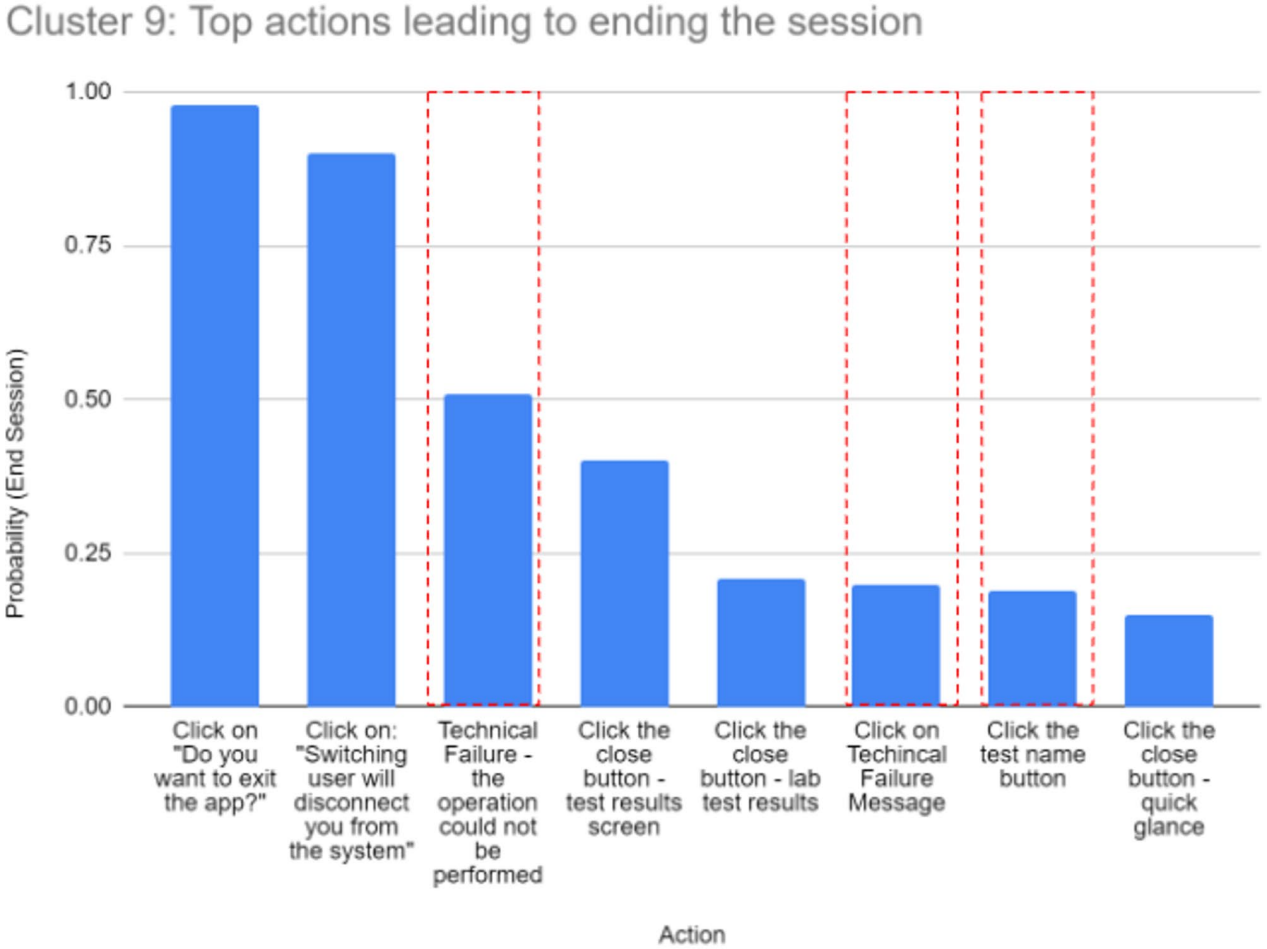
Cluster 9: Top actions leading to ending a session.

In this section, we leverage Markov models to identify pain points in the customer journey and, in particular, actions that lead to undesirable outcomes. We demonstrate the power of the Markov model in detecting actions that have a high probability of leading to churn (session ending). In the following example, we focus on cluster 9, which is related to journeys where customers view their laboratory test results. Figure 17 shows the top 8 actions with the highest transition probabilities for ending a session, according to a first-order Markov model.

The figure shows that the two most likely actions, “Clicking on: Do you want to exit the app?” and “Clicking on: Switching users will disconnect you from the system,” occurred exactly as expected. In contrast, some actions highlighted in red are not expected to cause a session to end. Two of these (the third and sixth from the left) are related to triggering a technical error, an issue that should be reviewed by a domain expert. Another action, namely, “clicking the button with the test name”, aims to provide more details about the meaning of the medical test type and is not expected to terminate the session. After investigation, it was found that clicking this button automatically redirected customers to an external website that contained the requested information. When customers aim to return to the mobile application, they have to close the website screen and load the mobile application screen. This process is likely the reason for the high percentage of customers who exit in the middle of the process without continuing to view their test results. One idea for improvement that comes to mind immediately is to display a popup with medical test information within the mobile application. In this way, interruption is prevented, and customers can continue navigating the app smoothly.

A related drawback that we encounter when using the first-order Markov model in this analysis is the lack of sufficient memory. For example, we know that a technical error has a high probability of causing a session to terminate, but this information is often useless, as we do not know what caused the error. In such a case, one could use a higher-order Markov model that captures more information about the pattern that leads to the error.

### Post cluster analysis

This part focuses on extending the cluster analysis phase and enriching it with customer data. In this analysis, we not only use event log data but also integrate customer/session characteristics. We demonstrate the importance of this step via an example. In particular, we focus on cluster 4, which is related to scheduling a doctor’s appointment. We use the ages of the customers to distinguish between two segments of customers: customers aged 55 years and below and customers over 55 years old.

**Fig. 18:**
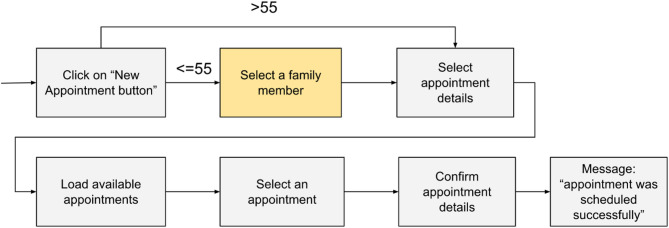
Cluster 4: Scheduling a doctor appointment—most representative paths: Customers aged 55 years and younger versus customers over 55 years.

Figure 18 shows the most representative paths in this cluster for each age segment. The representative paths are quite similar, except for one action, which is highlighted in yellow.

This action, “select a family member”, is used to change the customer to whom the service is provided; this means that customers up to 55 years old are more likely to schedule appointments for their family relatives (often children), whereas customers over 55 years old are more likely to schedule appointments for themselves.

## Discussion, contributions and future work

The exponential growth in available data from digital event logs alongside the growing importance of excellent service levels has led researchers and practitioners to focus on the importance of the digital customer journey. In addition to service quality, analyzing customers’ digital behavior helps reduce operating costs, improve digital apps and better segment and personalize services. This can be achieved by encouraging more customers to use digital rather than physical services, thus saving human resources while recording and analyzing customer behavior digitally. As a result, from a business perspective, using such data improves personalization and marketing activities based on customer segments and behavior patterns.

The purpose of this study was to address a clear gap in the literature and to respond to the need for a systematic method for identifying customers’ digital behavioral patterns with minimal manual intervention and adjustments. Previous work focused on visualizing the customer journey. This work addresses this gap by proposing a systematic methodology for analyzing customer journeys to derive actionable insights that can be used to improve customer experience, engagement and satisfaction, particularly in high-stakes domains like healthcare.

This research offers three main contributions. First, we propose a reproducible analytic framework that combines widely used methods—topic modeling (LDA/hLDA) and Markov chain analysis—to analyze digital customer journeys at multiple granularity levels and to mitigate the ‘spaghetti model’ challenge via principled session summarization and cluster-specific sequence modeling. Second, using a unique real-world smart-health event-log dataset of a leading HMO, we demonstrate how the framework can be implemented at scale to extract actionable insights relevant to digital healthcare services. Third, we show how the framework supports practical diagnostic tasks (e.g., representative-path extraction, detection of transitions associated with undesirable outcomes, and identification of ambiguous sessions/leakage between journey types) that are difficult to obtain from visualization-only approaches.

By separating co-occurrence-based clustering from transition-based modeling, the framework avoids the exponential state-space growth that would arise if full sequence information were incorporated directly into topic modeling. The Markov stage explicitly captures temporal order, enabling the identification of representative flows, high-probability transitions, and actions associated with undesirable outcomes.

While our case study is grounded in a healthcare mobile application, the framework is domain-agnostic in that it only assumes the availability of session-based event logs and discrete user actions. In e-commerce, banking, or e-learning platforms, the same pipeline, i.e. the session aggregation, topic-based clustering via LDA or hLDA, and per-cluster Markov modeling, may be applied with minimal changes, mainly in the selection of filtering thresholds and the definition of domain-specific ‘stop actions’ that are removed to reduce noise. Adding such domain-tailored preprocessing rules allows the framework to generalize to a wide range of digital customer journey settings.

While the current evaluation is limited to a single real-world healthcare mobile app dataset, mainly due to data availability constraints, the framework’s core components, LDA for topic-based clustering and Markov chains for sequential modeling, are standard, domain-agnostic techniques widely applied across diverse fields such as e-commerce, web analytics, and sentiment analysis. Despite the fact that cross-domain tests were not conducted, the method’s reliance on generic event log preprocessing (e.g., session aggregation and action filtering) and unsupervised modeling suggests strong potential for transfer to other digital platforms, such as banking apps or e-learning systems, with only minor adjustments to filtering thresholds.

Several research directions can be considered for applying the proposed framework to different data and/or different objectives. An interesting future direction could be to evaluate how the use of (h)LDA as a preliminary step for session clustering before creating the Markov models can contribute to the performance of predicting the next step in the customer journey, compared with the use of Markov models as a stand-alone technique^19^. Such an approach could be useful for companies that want to increase their conversion rates, such as e-commerce companies and CRM modules. The proposed framework could also be used for link optimization and can extend the work of Postelnicu et al.^53^, who presented an algorithm based on Markov models for shortening the expected path length on a website. Performing such optimization over the topics obtained by (h)LDA separately, rather than over all available routes, could result in a substantial reduction in the number of steps, leading to a better customer experience. Another important extension of this work would be the incorporation of sequence-aware topic models, such as Dynamic Topic Models or Hidden Markov Topic Models, which jointly model co-occurrence and temporal dependencies. Additionally, mixture-of-Markov models or fully Bayesian hierarchical sequence models may provide a unified probabilistic treatment of topic uncertainty and transition dynamics. Moreover, as digital systems evolve, new features introduce new actions, and UX changes can shift navigation patterns. Our framework can be applied in a *time-sliced* or *version-conditioned* manner using release metadata (e.g., system version). Practically, one can run the pipeline separately per version and quantify drift via distributional changes in (*i*) topic–action distributions and (*ii*) cluster-specific transition matrices. When drift exceeds a predefined threshold, the topic model and Markov models are re-estimated (or incrementally updated), enabling the framework to adapt to new behaviors while preserving interpretability across releases.

In addition, our framework currently supports the analysis of digital customer journeys^3^ on a single platform. As an increasing number of companies offer multichannel services, it would be beneficial to extend the proposed approach to a multichannel service environment while considering dwell times, which are meaningful and can provide more actionable insights. Finally, a natural extension of the presented fixed-order Markov models is a nonhomogeneous VOBN^54^. With this extension, we can derive meaningful sequential behaviors while maintaining relatively low space complexity. Moreover, while our empirical study focuses on discrete textual event logs, many customer journeys are captured in multimodal form (e.g., screen recordings, support call audio, short videos, or screenshots)^55^. A practical extension is to convert modality-specific signals into *time-stamped*,* discrete events* aligned to the session timeline, and then reuse the same pipeline to extract tokens/events from each modality (e.g., OCR, ASR, or video-derived events), or merge them with the clickstream as an augmented event sequence. Then, one can apply (h)LDA for session-level summarization and Markov models for sequential dynamics. This can enrich the interpretation of friction points by adding signals that are not visible in clickstream logs alone (e.g., inferred frustration during a repeated loop). Such extensions also raise privacy and governance considerations, especially in healthcare, that could be addressed via appropriate anonymization and access controls.

In summary, this study introduces a scalable, data-driven framework that combines LDA/hLDA topic modeling with Markov chains to uncover actionable patterns in digital customer journeys, demonstrated effectively on a large-scale healthcare dataset. By simplifying complex event logs into interpretable clusters and sequences, the approach enables organizations to enhance user experience, detect inefficiencies, and drive customer engagement and loyalty, offering a reproducible tool with broad applicability beyond healthcare.

### The emerging role of AI and large language models (LLMs) in digital customer journey analysis

Recent advances in Artificial Intelligence (AI), particularly the development of large language models (LLMs), have begun reshaping the digital behavior analytics and customer journey mapping domains. These models affect two major shifts in traditional methods. First, natural language understanding; Second, the ability to generate context-aware, human-like interpretations of complex event sequences^20,56^.

While our framework uses topic modeling and Markov models to extract patterns from structured session data, LLMs can be deployed to augment these findings by interpreting unstructured user-generated content (e.g., free-text inputs, chat logs, feedback). This enables the detection of nuanced intentions behind behaviors that structured logs alone may not capture. For example, combining LLMs with behavioral sequences may assist to infer latent dissatisfaction even in sessions that do not end in technical errors or abandonment.

Additionally, generative capabilities for customer journey optimization may improve digital pathways by simulating diverse user personas and predicting potential journey bottlenecks. For example, an LLM could be prompted with a specific demographic profile and asked to simulate a session flow through an application, identifying where users might get confused, frustrated, or exit prematurely. This complements our Markov-based detection of undesirable outcomes by providing proactive design recommendations. Moreover, when paired with federated learning^57^, or causal inference methods, LLMs could be used to propose interventions—such as notifications or layout changes—that are more likely to lead to successful outcomes. This level of integration opens pathways for semi-automated optimization of digital customer journeys in real time. Finally, the framework’s explainability facilitates integration with AI systems like LLMs. LDA/hLDA yields interpretable action clusters via topic distributions and word clouds, while Markov chains provide transparent transition probabilities. This supports traceable insights for decision-making in healthcare^58^.

As healthcare services become increasingly personalized and data-intensive^59,60^, integrating LLMs into customer journey analytics offers promising opportunities for real-time insights, anomaly detection, and personalized digital support. Future iterations of our framework may incorporate LLMs to enhance interpretability and to support more intelligent, adaptive user interface design in healthcare platforms.

## Data Availability

The datasets used and analyzed during the current study are available from the corresponding author upon reasonable request.
